# Pericytes Promote More Vascularization than Stromal Cells via an Interleukin‐6‐Dependent Mechanism in Microfluidic Chips

**DOI:** 10.1002/advs.202408131

**Published:** 2025-01-30

**Authors:** Julian Gonzalez‐Rubio, Hannah Kubiza, Yong Xu, Hiltrud Koenigs‐Werner, Mona Sophie Schmitz, Michaela Schedel, Christian Apel, Stefan Jockenhoevel, Christian G. Cornelissen, Anja Lena Thiebes

**Affiliations:** ^1^ Department of Biohybrid & Medical Textiles (BioTex) AME – Institute of Applied Medical Engineering Helmholtz Institute RWTH Aachen University Forckenbeckstrasse 55 52074 Aachen Germany; ^2^ Institute of Pathology Electron Microscopy Facility RWTH Aachen University Hospital Aachen, Pauwelsstrasse 30 52074 Aachen Germany; ^3^ Department of Pulmonary Medicine University Medicine Essen‐Ruhrlandklinik Tueschener Weg 40 45239 Essen Germany; ^4^ Department of Pulmonary Medicine University Medicine Essen Essen, Hufelandstraße 55 45147 Essen Germany; ^5^ Clinic for Pneumology and Internal Intensive Care Medicine (Medical Clinic V) RWTH Aachen University Hospital Pauwelsstrasse 30 52074 Aachen Germany

**Keywords:** angiogenesis, blood vessels, endothelial cells, mesenchymal stem cells, organ on a chip

## Abstract

Pericytes are a key player in vascularization, protecting endothelial cells from external harm and promoting the formation of new vessels when necessary. However, pericytic identity and its relationship with other cell types, such as mesenchymal stromal/stem cells, is highly debated. This study compares the role of pericytes and unselected stromal cells in vascularization using multichannel microfluidic chips. In both angiogenesis and vasculogenesis, pericytes promote more vessel formation than stromal cells. Pericytes can wrap around endothelial vessels acting as mural cells, while stromal cells remain separated. Whole‐transcriptome sequencing confirms an upregulation of pro‐vascularization genes in endothelial cell‐pericyte co‐cultures, while metabolism increases and inflammation decreases in stromal cell co‐cultures. Treatment of stromal‐endothelial cell co‐cultures with either conditioned media or isolated extracellular vesicles from pericytes replicates the increase in vasculogenesis of the direct co‐cultures. Cytokine quantification reveals that interleukin 6 (IL‐6) is significantly increased in pericyte conditions. Blocking it with siltuximab results in a reduction of pericyte vasculogenic potential comparable to stromal cell levels, revealing that pericyte pro‐vascularization is mediated by IL‐6. This study provides new insights into the relationship between pericytes and endothelial cells and the elusive identity of mesenchymal stromal cells. These findings are relevant for both vascular biology and tissue engineering.

## Graphical abstract

This study shows how selected pericytes outperform stromal cells in promoting angiogenesis as well as vasculogenesis in a microfluidic organ‐on‐a‐chip platform and by whole mRNA sequencing. It demonstrates how pericyte extracellular vesicles, and more specifically interleukin‐6, mediate this angiogenic stimulation. These findings shed light on one of the biggest tissue engineering challenges, achieving vascularization in vitro.

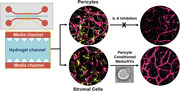

## 1 Introduction

The circulatory system is the interconnected and hierarchical network of vessels that irrigate most human tissues, nourishing the cells with nutrients and oxygen, removing waste products, and serving as a connection for the endocrine and immune systems to swiftly reach any part of the body.^[^ ^1^ ^]^ All vessels are lined with endothelial cells. In the capillaries, the smallest branch of the circulation, endothelial cells are lined externally by a mural cell type known as pericyte, which supports their homeostasis, protects them from stromal debris by phagocytosis, and regulates blood flow.^[^ ^2^ ^]^

For tissue engineering, the size of non‐vascularized bioengineered organs is limited to a few hundred micrometers.^[^ ^3^ ^]^ Any thicker tissue without irrigation of a blood‐mimicking fluid will eventually lead to cell death due to the lack of oxygen and nutrient diffusion to the innermost parts, causing the formation of a necrotic core. However, achieving vascularization in in vitro systems is still a challenge due to the complexity of the process and the variety of cells involved.^[^ ^4^ ^]^ The two main processes driving neovascularization are vasculogenesis, in which endothelial cells organize themselves into interconnected vessels, and angiogenesis, in which new vessels originate from pre‐existing ones.

Human adipose tissue is an easily obtainable biological source, which can be digested using collagenases to extract a heterogeneous mixture of cells, known as stromal vascular fraction (SVF).^[^ ^5^ ^]^ The spindle‐shaped cells produced by seeding the SVF on cell culture‐treated plastic are often referred to as adipose‐derived mesenchymal stromal/stem cells or adipose stromal cells^[^ ^6^ ^]^ (further referred to as stromal cells). They have been widely reported to have immunomodulatory properties^[^ ^7^ ^]^ and in vitro multipotency.^[^ ^8^ ^]^ Within this heterogeneous collection, we can find a wide variety of stromal cells such as fibroblasts, pre‐adipocytes, and the previously mentioned pericytes.^[^ ^5^ ^]^ The melanoma cell adhesion molecule, also known as cluster of differentiation (CD) 146, can act as a mural cell marker in bone marrow‐derived stromal cells^[^ ^9^ ^]^ and has been proposed as a specific membrane protein for adipose‐resident pericyte isolation.^[^ ^10^ ^]^ Importantly, CD146 is not exclusive to pericytes and can be expressed in other vascular‐related cell types such as lymphocytes and endothelial cells, but these cells can be depleted due to their incapability or inefficiency to attach and divide on uncoated hydrophilic plastic surfaces.^[^ ^11^ ^]^ The identity of pericytes in vivo and, especially, in vitro, is however widely debated,^[^ ^12^, ^13^ ^]^ as well as their relationship to mesenchymal stem cells, their contribution to vascularization processes, and their use in cell therapies and tissue engineering.^[^ ^14^, ^15^, ^16^ ^]^

In recent years, microfluidic devices allowed the creation of organ‐on‐chip technologies, also known as microphysiological systems.^[^ ^17^ ^]^ These platforms have been demonstrated as powerful tools for the study of tissue development, cell‐cell and cell‐matrix interaction, and vascularization.^[^ ^18^, ^19^ ^]^

Here, we use a microfluidic platform to mimic vasculogenic and angiogenic processes, comparing how pericytes and stromal cells interact with endothelial cells to promote the formation of vessels. The present study is based on two hypotheses, 1) that CD146 serves as a reliable marker for the selection of pericytes from heterogeneous adipose stromal cells, and 2) that pericytes can be cultured in vitro without losing the capacity to act as mural cells, promoting vessel formation better than their unselected counterpart and associating with the endothelial cells by a shared basement membrane. We investigate these assumptions at both the cellular and molecular levels using confocal and electron microscopy, as well as transcriptomic analysis. We further isolated, characterized, and tested the pro‐vasculogenicity of conditioned media and extracellular vesicles (EVs) from both types of supporting cells. Finally, we assessed if interleukin‐6 is responsible for the increased vascularization promoted by the pericytes by blocking it with the monoclonal antibody siltuximab.

## 2 Results and Discussion

### 2.1 Microfluidic Chip Platform to Study Vessel Formation via Vasculogenesis and Angiogenesis

**Figure 1:**
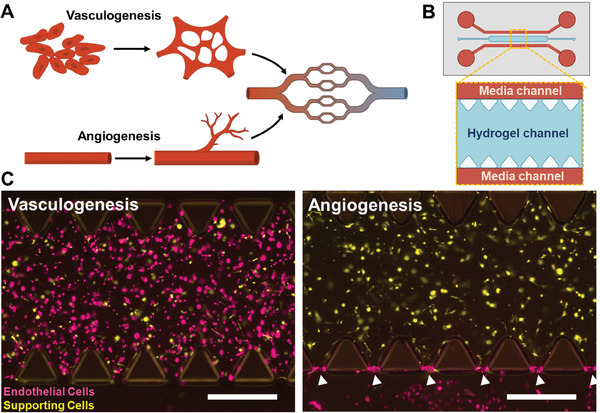
Replication of the two main vascularization processes in microfluidic devices. A) Main mechanisms of vessel formation in the human body. B) A schematic drawing of the microfluidic chip shows the internal hydrogel channel (blue) and the surrounding parallel media channels (red). C) Fluorescence images showing freshly seeded endothelial cells (Vybrant DiD, magenta) and supporting cells (Vybrant DiO, yellow), which can be either pericytes or stromal cells, in two different set‐ups to study vasculogenesis (left) and angiogenesis (right). White arrows mark the interface between the hydrogel and media channel where the endothelial cells attach. Scale bars: 500 µm.

To compare the processes of vasculogenesis and angiogenesis (**Figure** **1**A), and how pericytes and stromal cells act in both, we used a commercially available three‐channel microfluidic chip from AIM Biotech, Singapore (Figure 1B). To mimic vasculogenesis, human umbilical vein endothelial cells (HUVECs) and supporting cells – hereafter used as a collective term for adipose pericytes and stromal cells – are both added to the fibrin hydrogel in the middle channel in a 5:1 ratio. The endothelial/supporting cell ratio was chosen based on previous work on the same commercial chip^[^ ^18^, ^20^ ^]^ and aligns with the range observed in vivo.^[^ ^21^ ^]^ The other two parallel channels are used as media reservoirs. To investigate sprouting angiogenesis, on the other hand, only supporting cells are added in the hydrogel middle channel and one of the two media channels is coated with endothelial cells (Figure 1C). Then, the media in the channel opposite to the endothelialized one (which does not have cells and is solely used as a media reservoir) is supplemented daily with 50 ng mL^−1^ VEGF, as an exogenous stimulus necessary to promote the invasion of endothelial cells into the gel.^[^ ^22^ ^]^ In both setups, the chips are incubated for ten days with daily media changes.

The pre‐staining of the cells with lipophilic dyes confirms the deposition of the cells in the hydrogel after injection. 24 h after the preparation, endothelial cells attached to the hydrogel‐media interface in the angiogenesis condition, marking the beginning of angiogenesis (Figure 1C, right image). The pericyte and stromal cell co‐cultures were incubated for ten days and stained with calcein AM and propidium iodide to assess whether cells survived within the chips, resulting in high survival rates of 95% and 93%, respectively (Figure S1, Supporting Information).

### 2.2 CD146‐Enriched Stromal Cells can Act as Mural Cells In Vitro and Show Enhanced Pro‐Angiogenic Potential

**Figure 2:**
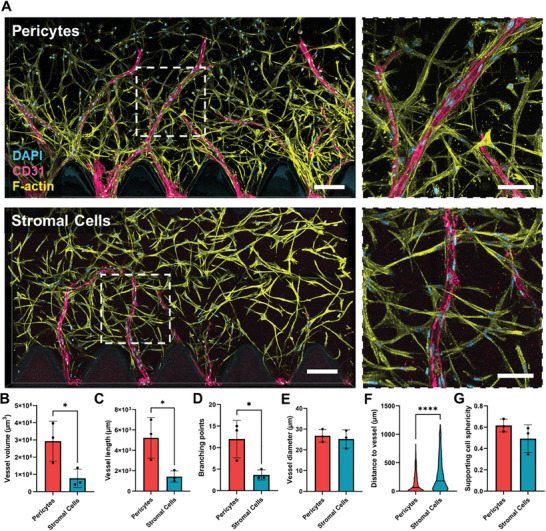
Comparison of the pro‐angiogenic potential of pericytes and stromal cells. A) Immunofluorescence images of the angiogenic sprouting within the microfluidic chips co‐cultured with either pericytes or stromal cells, stained for nuclei (DAPI; cyan), CD31 showing the endothelial vessel‐like structures (magenta) and the supporting cells’ F‐actin cytoskeleton (Phalloidin; yellow). F‐actin is not visible within the endothelial cells since it was subtracted from the picture during the image processing to increase clarity. Scale bar: 200 µm (left), 100 µm (right). B–E) Comparison of total vessel volume (*p* = 0.0428), length (*p* = 0.0340), branching points (*p* = 0.0329), and average diameter (*p* = 0.6508) between both co‐culture conditions (n = 3, unpaired t‐test, *p *< 0.05 is considered significant). F) Comparison of the distance between supporting cells and the nearest vessel‐like structure (*p* < 0.0001). The trunked violin plots depict summary statistics and the kernel density estimation to show the frequency distribution of each condition. The middle line represents the median (n = 3, Mann‐Whitney test, *p *< 0.05 is considered significant). G) Comparison of supporting cells’ average sphericity (*p* = 0.2667, n = 3, unpaired t‐test, p < 0.05 is considered significant). Pericytes and stromal cells from 3 different donors each were used for the co‐cultures (n = 3), while the endothelial cell donor remained constant. Asterisks indicate a statistical significance (*p < 0.05, ****p < 0.0001).

We separated pericytes from the heterogeneous adipose SVF based on the expression of the CD146 marker using immunomagnetic microbeads. Pericytes and unselected stromal cells were cultured in mesenchymal cell culture media and expanded for up to four passages. To investigate how these cell types compare to each other in promoting angiogenesis, each cell type was co‐cultured with endothelial cells in microfluidic devices (n  =  3 independent donors, Figure 1C, right). The co‐cultures were maintained for ten days, fixed, and stained for the endothelial marker CD31, and F‐actin to visualize supporting cells. Confocal microscopy shows that pericytes promoted more vessel sprouting than stromal cells (**Figure** **2**A). Statistically significant differences can be found in total vessel volume, length, and branching points (Figure 2B–D, details on image processing and quantification can be found in Figure S2, Supporting Information and in Section 4.5). Pericytes have previously been reported to be highly pro‐angiogenic supporting cells due to their close relation with endothelial cells in vivo.^[^ ^9^, ^23^, ^24^ ^]^ Interestingly, increased angiogenesis is not correlated with any remarkable difference in vessel diameter (Figure 2E). Vessel lumen could not be confirmed or measured due to limitations in the confocal microscopy resolution. As observed in the higher‐magnification immunofluorescence images, pericytes tend to be significantly closer to or even directly associated with the vessels, while the stromal cells remain homogenously distributed in the gel (Figure 2A,F). This interaction is expected from pericytes, which are known to wrap around blood vessels in vivo to support and regulate their functions.^[^ ^20^ ^]^ This way, pericytes can exert direct cell‐cell pro‐angiogenic stimulation and stabilize newly formed vessels.^[^ ^21^ ^]^ Apart from their relative position, pericytes do not show differences in sphericity compared to the stromal cells (Figure 2G). The media from day 10 was analyzed with flow cytometry‐based cytokine quantification. Interestingly, none of the ten molecules highly related to angiogenesis were more present in the media of the pericyte co‐cultures (Figure S3, Supporting Information). This could be caused by different reasons, such as growth factors being bound to their receptors and not present freely in the media^[^ ^22^ ^]^ or as in the case of the pericytes, that the pro‐angiogenic regulation is directly performed cell‐to‐cell by integrins and other adhesion molecules, taking advantage of the closeness of the mural cells to the endothelium.^[^ ^23^ ^]^ Angiopoietin 2 levels were so high in both conditions that the upper detection limit of the assay (100 000 pg mL^−1^) was reached. VEGF is known to be an activator of angiopoietin 2 expression, and the angiogenesis assay requires the addition of a high amount of this factor in the media opposite to the endothelialized channel, which might play a role in this generalized high presence.^[^ ^25^, ^26^ ^]^ Overall, we can confirm that the CD146‐selected pericytes maintain their pro‐angiogenic activity and identity in vitro and perform better than the unselected counterparts.

### 2.3 Pericytes Promote More Vessel Formation during Vasculogenesis Than Stromal Cells

**Figure 3:**
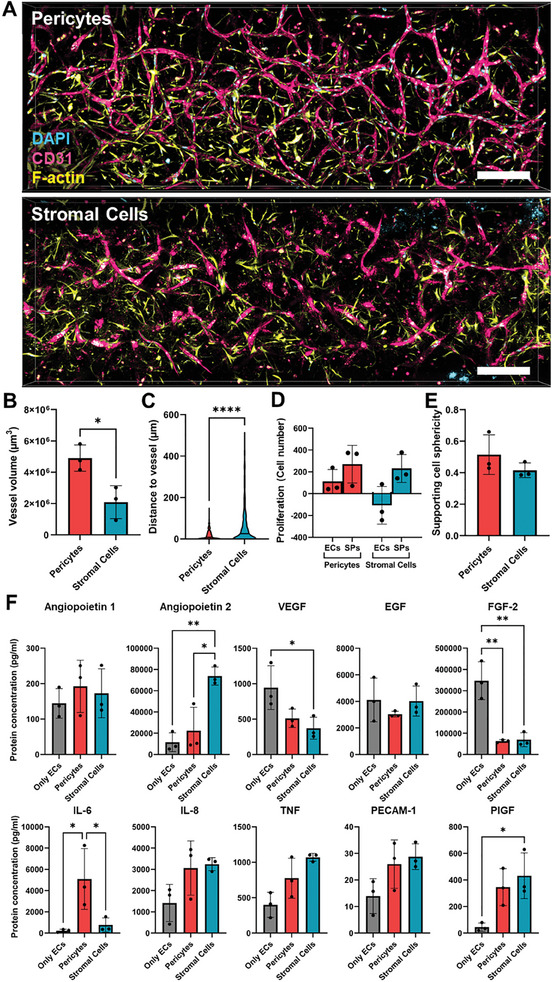
Comparison of pericytes and stromal cells in promoting vasculogenesis of endothelial cells. A) Immunofluorescence images of the vasculogenesis process within the microfluidic chips co‐cultured with either pericytes or stromal cells, stained for nuclei (DAPI; cyan), CD31 showing the endothelial vessel‐like structures (magenta) and the supporting cells’ actin cytoskeleton (F‐actin, Phalloidin; yellow). Scale bars: 200 µm. B) Comparison of total vessel volume between both co‐culture conditions (*p* = 0.0223, n = 3, unpaired t‐test, *p *< 0.05 is considered significant). C) Comparison of the distance between each type of supporting cell and the nearest vessel‐like structure. The trunked violin plots depict summary statistics and the kernel density estimation to show the frequency distribution of each condition. The middle line represents the median (n = 3, Mann‐Whitney test, *p *< 0.05 is considered significant). D) Comparison of the final total number of endothelial cells (ECs; *p =* 0.1372, n = 3, unpaired t‐test, *p *< 0.05 is considered significant) and supporting cells (SPs; *p =* 0.999, n = 3, Mann‐Whitney test, *p *< 0.05 is considered significant). E) Comparison of supporting cells’ average sphericity (*p* = 0.2099, n = 3, unpaired t‐test, *p* < 0.05 is considered significant). F) Quantification and comparison of ten cytokines tightly related to vascularization, namely angiopoietin 1, angiopoietin 2, vascular endothelial growth factor (VEGF), epidermal growth factor (EGF), fibroblast growth factor 2 (FGF‐2), interleukin‐6 (IL‐6) and interleukin‐8 (IL‐8), tumor necrosis factor (TNF), PECAM‐1 and placenta growth factor (PlGF). Statistical values can be found in Table S1 (Supporting Information) (n = 3, *p *< 0.05 is considered significant). Pericytes and stromal cells from 3 different donors each were used for the co‐cultures (n = 3), while the endothelial cell donor remained constant. Asterisks indicate statistical significance (*p < 0.05, **p < 0.01, ****p < 0.0001).

Immunofluorescence images of the microfluidic device comparing pericytes and unselected stromal cells in vasculogenesis (Figure 1C, left) show clear differences in how both cell types support endothelial cell coalescence and elongation (**Figure** **3**A). Pericytes promote the formation of longer and more interconnected tubular endothelial structures, while co‐culture with stromal cells leads to the formation of less and more disconnected structures. In comparison, endothelial cells alone are unable to form any organized structure, confirming that supporting cells are essential for vasculogenesis (Figure S4A, Supporting Information). Quantification of the vessel‐like structures shows a significant increase in vessel volume in the samples co‐cultured with pericytes (Figure 3B). As in angiogenesis, pericytes are significantly closer to the vessels than the stromal cells, suggesting a mural cell activity (Figure 3C). We quantified cell proliferation by comparing the final number of endothelial and supporting cells to the amount seeded at the beginning of the experiment (Figure 3D; Figure S4B, Supporting Information), and no significant difference was found between the two conditions. The difference in morphology between the two supporting cell types was also not significant (Figure 3E).

Most vasculogenic processes are started and guided by extracellular signaling. Therefore, we quantified the cytokine concentration in the last days’ medium of the co‐cultures and of the endothelial cells alone as control (Figure 3F). Out of the ten measured cytokines, interleukin 6 (IL‐6) was the only one that was significantly more present in the media of the pericyte co‐cultures, with a six‐fold increase in comparison to the stromal cells. IL‐6 strongly stimulates the production of VEGF and hypoxia‐inducible factor 1‐alpha (HIF‐1α), two of the most potent pro‐angiogenic molecules.^[^ ^27^ ^]^On the other hand, angiopoietin 2 is significantly higher in stromal cells than in the other two conditions. Angiopoietin 2 is known to have an ambivalent role in vascularization, depending on VEGF concentration. With high VEGF levels, angiopoietin 2 and VEGF act synergically to promote endothelial cell growth and migration. However, with lower VEGF levels, angiopoietin 2 acts as an anti‐angiogenic molecule by binding and blocking the angiopoietin 1 receptor TIE2.^[^ ^25^ ^]^ When compared to endothelial cells alone, the stromal cell co‐cultures also stand out for the higher concentration of PECAM‐1 and PlGF. Curiously, both VEGF and FGF‐2 are more present in the media of the endothelial cells alone than in the pericytes and stromal cells.

Overall, we show that pericytes promote the formation of bigger and more interconnected vessels than stromal cells, and that this activity might be mediated by increase of IL‐6 and decrease of angiopoeitin‐2.

### 2.4 Pericytes But Not Stromal Cells can Act as Mural Cells for Endothelial Vessels

**Figure 4:**
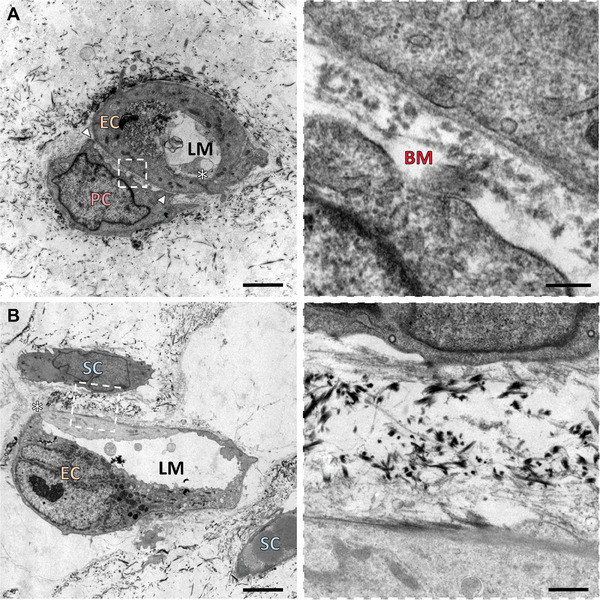
TEM image of the contact between supporting cells and vessels (A: pericytes, B: stromal cells). Only pericytes acting as mural cells possess a common basement membrane matrix with the endothelial cells. EC: Endothelial Cell; PC: Pericyte; SC: Stromal Cell; BM: Basement membrane; LM: Lumen; Arrows: pericyte‐endothelial cell junction; *: endothelial junction. Scale bars: 2500 nm (top left), 5000 nm (bottom left), 250 nm (right).

The main identifier of mural cells in vivo is the presence of a dense fiber network shared with the endothelial vessels, known as the basement membrane.^[^ ^23^ ^]^ Confirming the previous findings, transmission electron microscopy (TEM) reveals a close association of many pericytes with the endothelial vessels, forming a common basement membrane of shared matrix fibers (**Figure** **4**A). Such interaction is rarely observed between stromal cells and endothelial cells, which only present a loose association without a remarkable amount of shared fibers (Figure 4B). The incubation of the samples with tannic acid, which stains collagen and elastin in black, reveals that the composition of both matrices is also distinct. The space between stromal and endothelial cells is filled with dark fibrous collagen and elastin, while the basement membrane remains mostly unstained. The basement membrane is a combination of laminin, collagen IV, fibronectin, and many other proteins, explaining the gray tone of the interface.^[^ ^23^ ^]^ The basement membrane is interrupted by contact zones between the mural cell and the endothelial cell membranes (Figure 4A, arrows), allowing direct cell‐cell communication through integrins and other transmembrane molecules.^[^ ^24^ ^]^ The areas in which the endothelial cells attach to themselves or others by tight junctions, such as claudin‐5, or by adherens junctions, such as CD31 and VE‐cadherin, are also visible in the TEM images (Figure 4A,B, asterisks).^[^ ^28^ ^]^ Unlike the previous experiments, TEM was performed on bulk gels molded in well plates instead of microfluidic chips, since removing the gel from the microfluidic device without destroying it is not feasible.

### 2.5 A Transcriptomics Insight Into Supporting Cells’ Differential Behavior

**Figure 5:**
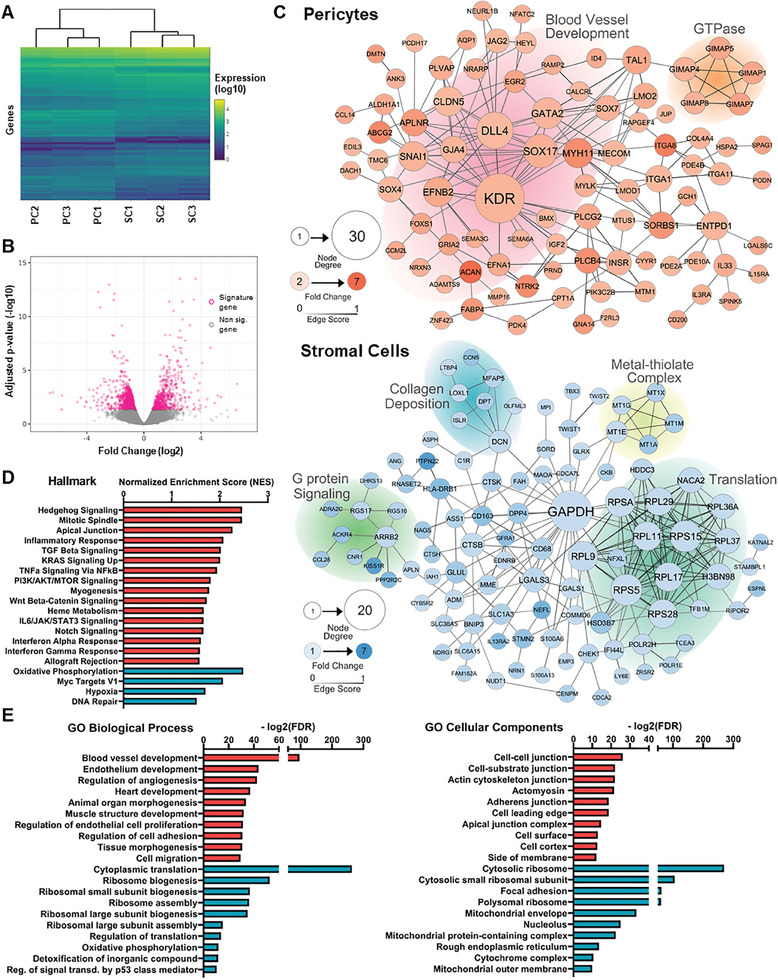
Gene expression analysis of pericyte and stromal cell co‐cultures with endothelial cells. A) Dendrogram and heat map of hierarchical clustering of the 1550 differently expressed genes between the three pericyte (PC) and stromal cell (SC) donors (n  =  3, for further explanations in the statistical analysis see the Methods section). B) Volcano plot of the 1550 differently expressed genes plotting their fold change (log2) by adjusted p‐value (‐log10). Positive fold change values are signature genes in pericytes, while negative ones are in the stromal cells. C) Network visualization of the top differently expressed genes in both types of co‐cultures. Each node represents a signature gene and the connections to other genes show a known relation in their expressions. The node color visualizes the fold change of its expression, and the size represents its importance in the network, understood as the number of relations with other genes. The tone of the connections shows the “Edge score”, the strength of the relation between them. The background‐shading groups are merely orientative and represent relevant gene sets based on the GO databases. D) Hallmark gene sets from MsigDB on the differentially expressed genes between pericytes (red) and stromal cell (blue) co‐cultures. The scale bar represents the Normalized Enrichment Score (NES). E) Ontological analysis of the differently expressed genes based on GO databases, of pericytes (red) and stromal cells (blue). The scale bar represents the adjusted False Discovery Ratio (FDR). Pericytes and stromal cells from 3 different donors each were used for the co‐cultures (n = 3), while the endothelial cell donor remained constant.

We used RNA sequencing (RNA‐seq) to understand the differences in vasculogenesis in terms of how pericytes and stromal cells differently regulate endothelial cell division and organization. Briefly, vasculogenesis gels with endothelial‐supporting cell‐co‐cultures incubated for ten days were minced and dissolved to isolate RNA and perform bulk 3′mRNA‐sequencing. Heatmap and dendrogram (**Figure** **5**A) show the 1550 significantly differently expressed genes (DEG) between the different supporting cell types (adjusted p‐value *p* < 0.05; raw data can be found at NCBIs Gene Expression Omnibus, http://www.ncbi.nlm.nih.gov/geo/, accession number: GSE287099). These DEGs are evenly distributed between both cell types (Figure 5B). To better visualize the most relevant results, the 200 signature genes of each co‐culture condition with the highest fold change were plotted in a network (Figure 5C) showing the relations (edges) between genes (nodes), removing the genes with no known interaction.

Gene set enrichment analysis (GSEA) of all DEG shows that pericytes promote the upregulation of genes related to blood vessel formation and organization (Figure 5D,E). No known signaling gene is exclusive for either adult angiogenesis or vasculogenesis, since both involve activation of the same pathways.^[^ ^29^ ^]^ Within them, Notch overexpression in the pericyte co‐culture is central to the regulation of endothelial cell behavior during neovascularization.^[^ ^30^ ^]^ The detection of free VEGFA by the KDR/VEGFR2 receptor activates an EC as a “tip cell”, suppressing its proliferation and driving it to lead vessel elongation. Delta‐like ligands (DLL4) on tip cells interact with Notch receptors (Notch1 and Notch4) on adjacent cells, inhibiting their tip cell behavior and marking them as “stalk cells”.^[^ ^31^ ^]^ This process is crucial to control tubulogenesis and avoid aberrant networks.

Elaborating on other upregulated pathways in pericytes according to the Hallmark analysis, sonic hedgehog (SHH) coordinates both embryological and post‐natal vascularization through both its canonical and non‐canonical pathways, promoting EC survival, differentiation, and maturation.^[^ ^32^ ^]^ SHH upregulates Notch1 in both endothelial cells and pericytes. The Wnt pathway, on the other hand, regulates vascularization through β‐catenin‐dependent mechanisms that affect cytoskeleton organization, directly influencing vessel tubulogenesis, remodeling, and stability.^[^ ^33^ ^]^ The formation of vessel lumen is dependent on mitotic spindle orientation since it will establish the apical‐basal polarity of the endothelial cells.^[^ ^34^ ^]^ The apex of the lateral membrane of these adjacent polarized endothelial cells is joined by apical junctions, which have a major role in regulating cell‐cell adhesion and paracellular permeability.^[^ ^35^ ^]^

The transforming growth factor beta (TGF‐β) pathway regulates pericyte attraction and activity and acts as a cytostatic by the inhibition of c‐Myc expression, stabilizing new vessels.^[^ ^36^ ^]^ Both phosphoinositide 3‐kinase‐AKT‐mammalian target of rapamycin (PI3K‐AKT‐mTOR) and Kirsten rat sarcoma virus (KRAS) are related pathways activated in endothelial cells by pro‐angiogenic molecules and act regulating the secretion of VEGF, nitric oxide and Angiopoietin 1/2.^[^ ^37^ ^]^

Consistent with the previously reported cytokine quantification, the RNA‐seq analysis shows upregulation of IL‐6 in pericyte co‐culture with respect to unselected stromal cells. The IL‐6 receptor is highly expressed in pericytes, and its binding activates the STAT3 transcription factor, increasing mural cell recruitment and the release of pro‐vasculogenic growth factors.^[^ ^27^, ^38^ ^]^ Other pro‐inflammatory cytokines such as tumor necrosis factor‐alpha (TNFa), interferon alpha and gamma, and the NF‐kB signaling pathways are also downregulated in stromal cells. Stromal cells are widely known to have immunomodulatory properties which are being explored in cell therapy.^[^ ^6^, ^7^, ^13^, ^15^, ^16^ ^]^ This is in line with the downregulation of many inflammatory molecules observed in that co‐culture condition.^[^ ^39^ ^]^

The GO Cellular Component analysis reveals overexpression of cell‐cell and cell‐matrix junctions in the pericyte co‐cultures, such as Claudin‐5 (CLD5) and many types of integrins (ITGA).^[^ ^28^, ^40^ ^]^ The tightness of the endothelial barrier is dependent on these cellular connections, essential to maintain the structural integrity of the vessels and avoid the leakage of the blood contents. Some of the upregulated integrins are also directly involved in vasculogenesis such as ITGA1 or ITGA11.^[^ ^40^ ^]^

Stromal cell co‐culture shows a robust upregulation of Myc targets and hypoxia pathways, both profoundly involved in cell metabolism and proliferation.^[^ ^41^ ^]^ Other pro‐mitotic signaling such as p53 and G protein cascades are also upregulated.^[^ ^42^, ^43^ ^]^ The consequences are shown by the increased cytoplasmic translation and ribosomal, mitochondrial, and nucleolar biogenesis.^[^ ^42^ ^]^ The increased DNA repair mechanisms and oxidative phosphorylation may be related to this increase in metabolic activity.^[^ ^44^ ^]^ Stromal cells also overexpress genes involved in collagen genesis and deposition, explained by the large percentage of fibroblasts present in this population.^[^ ^5^ ^]^

RNA‐seq was performed using bulk gels molded in 24‐well plates instead of microfluidic chips. The 10‐µL gel in the microfluidic chamber, containing ≈120 000 cells, is too close to the lower limit for sequencing. Furthermore, we wanted to avoid the extraction from inside the chip, a longer and more aggressive procedure, requiring first solving the gel completely before aspirating it, which is prone to losing a significant fraction of the RNA. This process could also alter the gene expression since changes in cells’ RNA transcription, such as an increase in stress and wound healing responses, can be observed within minutes after enzymatic digestion.^[^ ^45^ ^]^ For the same reasons, the cell content was not separated depending on cell type but sequenced at once. The RNAseq in gels is therefore limited by not being fully comparable to the microfluidic chips and its inability to discern between the effect of endothelial and supporting cells.

Not all differences in gene expression can be translated to protein amount and signaling since RNA‐seq is blind to post‐transcriptional regulation and protein stability and degradation. However, there is a very clear trend of upregulated vasculogenesis and tubulogenesis genes in the co‐cultures supported by pericytes, including Notch, TGF‐β, IL‐6, SHH, and Wnt. On the other hand, stromal cells lead to the overexpression of genes related to cell metabolism and organelle biogenesis (such as Myc and p53), together with the associated cell stress and the downregulation of inflammatory cytokines that characterize this population.

### 2.6 Pericyte‐Conditioned Media Increase Vascularization in Stromal‐Endothelial Cell Co‐Cultures

**Figure 6:**
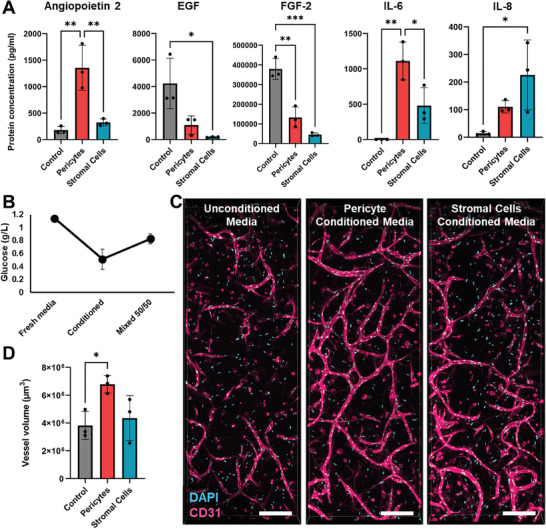
Analysis of conditioned media and effect on stromal cells’ vasculogenesis. A) Results of cytokine quantification in the media conditioned after 24 h of incubation with either an empty flask (control), pericytes, or stromal cells. Only results with significant differences are shown in this figure, cytokines with non‐significant differences can be found in Figure S5A (Supporting Information). Statistical values can be found in Table S2 (Supporting Information) (n = 3, *p *< 0.05 is considered significant). B) Quantification of glucose before and after 24 h of conditioning with the supporting cells, followed by the final 50/50 mixture of fresh and conditioned media used in further co‐culture experiments. C) Confocal imaging of the stromal‐endothelial cell co‐cultures treated with unconditioned (Control) and conditioned media from pericytes and stromal cells. Scale bars: 200 µm. D) Comparison of total vessel volume of Control versus Pericytes (*p* = 0.0465), Control versus Stromal Cells (*p* = 0.8455), and Pericytes versus Stromal Cells (*p* = 0.0938). (*F* = 5.559, *p* = 0.0431, n = 3, one‐way ANOVA, *p* < 0.05 is considered significant). The media was conditioned by pericytes and stromal cells from 3 different donors each (n = 3), while the stromal and endothelial cell donors of the co‐cultures remained constant. Asterisks indicate a statistical significance (*p < 0.05, **p < 0.01, ***p < 0.001).

We wanted to test if the factors released by the pericytes are sufficient for exerting this pro‐vasculogenic effect or if direct pericyte‐endothelial cell contact is required. For that, we started by conditioning fresh media by incubating it with pericyte and stromal cell mono‐cultures for 24 h. In the conditioned media, the pro‐angiogenic cytokines released were quantified. As a control, the normal supplemented media was incubated in an empty flask under the same conditions. Comparing these three conditions, both angiopoietin 2 and IL‐6 were significantly higher in the pericyte‐conditioned media than in the other two (**Figure** **6**A). The increase of IL‐6 is consistent with the vasculogenesis co‐cultures, but in this case angiopoietin‐2 has an opposite tendency. Most angiopoietin 2 is released by endothelial cells, which store high amounts of them in the Weibel‐Palade bodies and that are not included in the mono‐cultures.^[^ ^46^ ^]^ Consequently, this higher level is actually in the order of ten times lower than in the co‐cultures, making them not completely comparable. In contrast, IL‐8 was significantly higher in the stromal cell media. FGF‐2 was significantly lower in both supporting cell‐conditioned media, and EGF also in the stromal cell one; likely due to the high amount of receptors of both growth factors in connective tissue cells in general.^[^ ^47^ ^]^

Since both conditioned media showed a different cytokine composition, we investigated if supplementation of stromal cell co‐cultures with the pericyte‐conditioned media was able to replicate the increased vasculogenesis observed in the pericyte co‐cultures. Analysis of glucose levels in the conditioned media revealed a consumption of around half of the original amount (Figure 6B), which can be replenished to ≈0.8 g L^−1^ by mixing it 1:1 with fresh media. This reflects the standard procedure when culturing cells with conditioned media.^[^ ^48^ ^]^

Effectively, the addition of pericyte‐conditioned media to stromal‐endothelial cell co‐cultures during the ten days of incubation increased the formation of vessels significantly (Figure 6C,D), comparable to the direct pericyte‐endothelial cell co‐cultures (Figure 3B). Media conditioned by the stromal cells was also used, but its performance was not significantly different from either the pericyte or the unconditioned media, scoring slightly above the latter.

### 2.7 Pericyte Extracellular Vesicles (EVs) are more Homogeneous and Pro‐Vasculogenic than EVs from Stromal Cells

**Figure 7:**
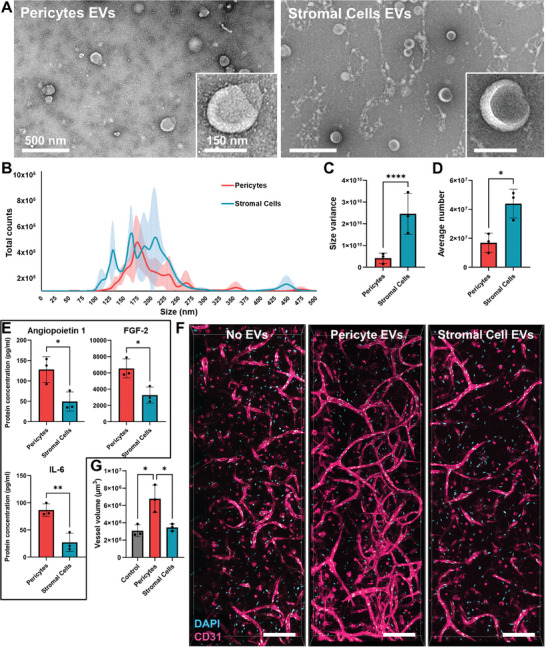
Characterization and vascularization potential of EVs from pericytes and stromal cells. A) TEM imaging of the EVs showing their “cup” shape. B) NTA measurement and quantification of the EVs from pericytes and stromal cells. C) Comparison of the EV size variance (*p* < 0.0001) between pericytes and stromal cells (n  = 3, F‐test, *p *< 0.05 is considered significant). D) Comparison of EV average number (*p =* 0.0177) between pericytes and stromal cells (n  = 3, unpaired t‐test, *p *< 0.05 is considered significant). E) Quantification and comparison of the EV cytokine content, specifically angiopoietin 1 (*p =* 0.0263), FGF‐2 (*p =* 0.0208) and IL‐6 (*p =* 0.0071). Only results with significant differences are shown in this figure, cytokines with non‐significant differences can be found in Figure S5B (Supporting Information). F) Confocal imaging of the stromal‐endothelial cell co‐cultures treated with the control (cell culture media treated in the same way as the conditioned media), and fresh media supplemented with EVs from pericytes and stromal cells. Scale bars: 200 µm. G) Comparison of total vessel volume in Control versus Pericytes (*p* = 0.0103), Control versus Stromal Cells (*p* = 0.8917), and Pericytes versus Stromal Cells (*p* = 0.0169). (*F* = 11.99, *p* = 0.008, n = 3, one‐way ANOVA, *p* < 0.05 is considered significant). The EVs were isolated from pericytes and stromal cells from 3 different donors each (n = 3), while the stromal and endothelial cell donors of the co‐cultures remained constant. Asterisks indicate a statistical significance (*p < 0.05, **p < 0.01, ****p < 0.0001).

A fundamental component of cell‐cell communication is EVs, and since they are highly studied and used in both in vitro and clinical settings, we focused on them as possible key players of the pericyte pro‐vasculogenicity. We isolated EVs from both pericyte and stromal cell‐conditioned media by filtration and a series of ultra‐centrifugation steps. Then, EVs were imaged with TEM to confirm their presence and state. EVs from both sources showed the characteristic “cup” shape acquired after the dehydration during the sample preparation (**Figure** **7**A). The EVs were quantified and measured using nanoparticle tracking analysis (NTA), revealing that stromal cells release significantly more vesicles, which also have a more heterogeneous profile, reflected by three main peaks at ≈130, 170, and 220 nm. Pericytes, on the other hand, have a more consistent EV production, peaking only at 180 nm (Figure 7B–D). The fact that EVs secreted by selected pericytes are more constant in size might derive from the selected pericytes being a more homogeneous population than the stromal cells.^[^ ^49^ ^]^

Then, EVs were treated with a lysis buffer to release their content and quantify the cytokines by a multiplex flow cytometry assay. A control was also prepared by treating fresh media in the same way as the conditioned one, expectedly resulting in cytokines below the assay's detection limit. IL‐6 was again significantly higher in the pericyte EVs, together with angiopoietin 1 and FGF (Figure 7E), while the other evaluated cytokines did not show any significant differences. Furthermore, it is worth noting that the concentration of IL‐8, TNF, and PlGF was so low in both EV types that can be considered almost negligible, which was not the case for the conditioned media. This suggests that only specific signaling molecules are loaded into EVs during normal cell secretion.

To assess the capacity of EVs to promote vascularization, they were resuspended in an equal volume of fresh, unconditioned media as the original conditioned media from which they were isolated. The EV‐supplemented media was then used to culture stromal‐endothelial co‐cultures for ten days, followed by vessel immunostaining and quantification. As with the conditioned media, the addition of pericyte EVs provoked a two‐fold increase in vessel volume, showing a significant difference to both the co‐cultures treated with the non‐EV media and the one with the stromal cell EVs (Figure 7F,G).

### 2.8 Blocking IL‐6 Equates the Pericyte's Pro‐Vascularization Potential to the Stromal Cells

**Figure 8:**
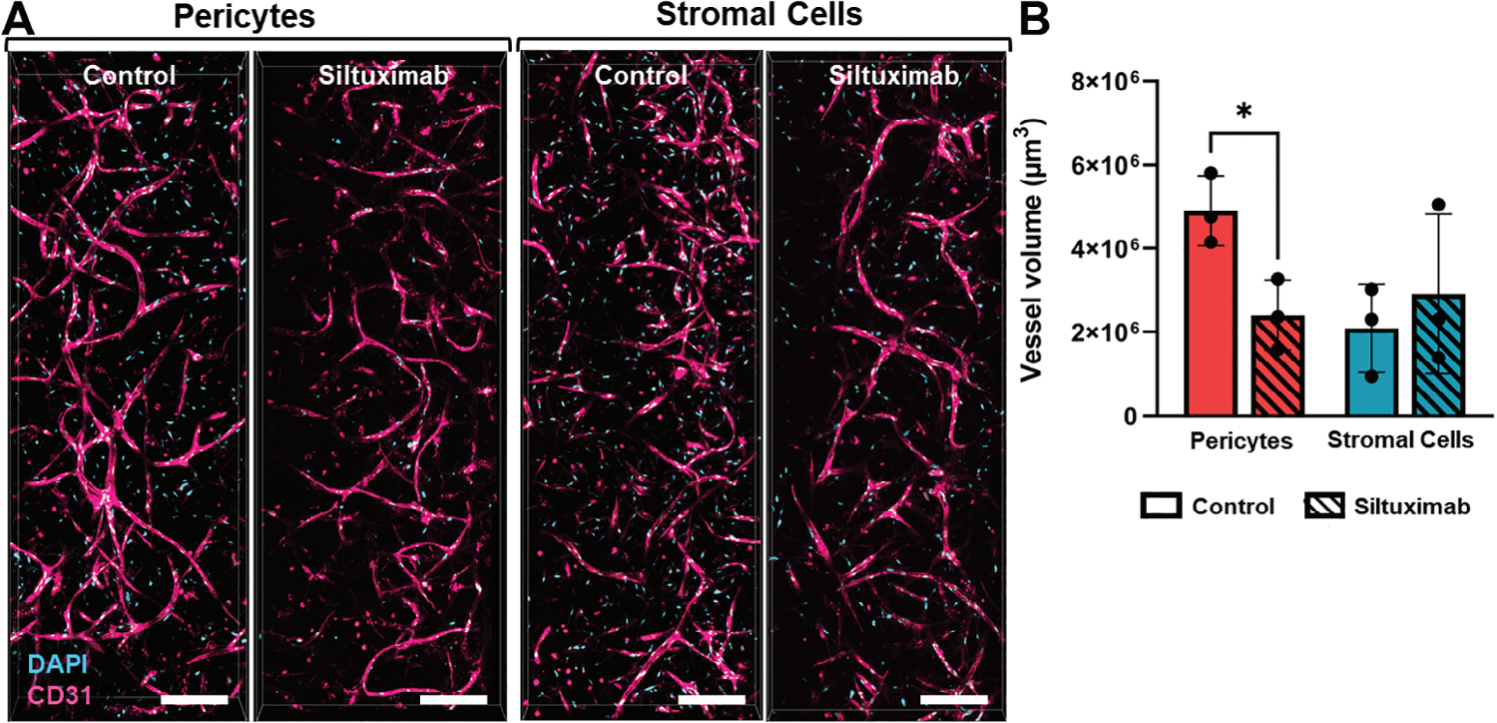
Effect of the IL‐6 inhibitor siltuximab in the pro‐vascularization potential of pericytes and stromal cells. A) Confocal images of the endothelial vessels (CD31, magenta) and nuclei (DAPI, clear blue) developed after co‐culturing them with pericytes and stromal cells, and with and without the addition of 100 ng/mL of siltuximab. B) Quantification and comparison of the vessel volume of the pericytes (*p* = 0.0215) and stromal cell (*p* = 0.5474) co‐cultures without and with siltuximab treatment (n = 3, unpaired t‐test, *p *< 0.05 is considered significant). Pericytes and stromal cells from 3 different donors each were used for the co‐cultures (n = 3), while the endothelial cell donor remained constant. Asterisks indicate a statistical significance (*p < 0.05).

In protein quantifications (Figures 3, 6, and 7D) and RNA expression analysis (Figure 5), IL‐6 consistently stood out with significantly increased levels in the pericyte conditions. Its mere increase, however, is not sufficient to tie it to the pericyte's greater vasculogenic potential. We decided to use the clinically‐approved monoclonal antibody siltuximab, an inhibitor of IL‐6, to assess whether this molecule is indeed a key mediator in the increased vascularization. The siltuximab concentration used was decided based on its binding stoichiometry with IL‐6 (1:1 molar ratio)^[^ ^50^ ^]^ and the amount of IL‐6 present in the media according to the previous analysis (≈5 ng mL^−1^, with a molecular weight ≈7 times lower than siltuximab), increasing it up to 100 ng mL^−1^ to account for undetected IL‐6 that was not measured because was bound and not free in the media. Endothelial cell co‐cultures with both pericytes and stromal cells were prepared to test the effect of siltuximab addition during the 10 days of culture in their vessel development. Supporting the previous observations, blocking IL‐6 led to a significant decrease in vascularization in the pericyte co‐cultures, diminishing their activity and equating them to the effect of unselected stromal cells (**Figure** **8**). As expected, the blocking IL‐6 in the stromal cell co‐cultures did not have any significant effect on their performance.

### 2.9 Limitations

Apart from previous limitations stated in the respective sections, other limitations of this study include the lack of in‐depth characterization of the heterogeneous adipose stromal cells. We refer readers to the comprehensive single‐cell study of Whytock et al. (2022) in that regard.^[^ ^51^ ^]^ Furthermore, even if RNA sequencing gives also a hint of highly expressed protein genes, only the ten most representative pro‐angiogenic cytokines were quantified in the culture media, thus risking overlooking additional molecules that could be relevant to the process. Finally, although pericytes are a rare population in the SVF (≈1%), the fact that the unselected stromal cells were not depleted from CD146+ cells leads to the possible inclusion of a small subpopulation of pericytes within them.

## 3 Conclusion

The relationship between pericytes and mesenchymal stromal cells has been widely debated in the last years, ranging from considering them as two completely independent populations to suggesting the latter as an activated form of the pericyte.^[^ ^9^, ^52^ ^]^ It has also been discussed if the pericytic identity can be maintained in culture or if subsequent passages in vitro would lead to a loss of its characteristics.^[^ ^9^ ^]^

Here, we demonstrate that the isolation of pericytes from the stromal vascular fraction of the adipose tissue based on the expression of the CD146 marker provides a population of plastic‐adherent cells that conserves their ability to act as mural cells in vitro for at least five passages. Both immunofluorescence and TEM imaging proved this interaction. We also demonstrate that these pericytes act significantly differently from their unselected counterpart, promoting longer and more branched vessels in both angiogenesis and vasculogenesis. This is also reflected in the genome‐wide expression analyses, which show that pericytes upregulate pathways related to vessel formation, such as VEGFA‐VEGFR‐DLL4‐Jagged1‐Notch, SHH, Wnt, IL‐6‐JAK‐STAT3 and PI3K‐AKT‐mTor; while stromal cells stimulate cell metabolism and biogenesis through pathways associated to Myc and p53, and decrease pro‐inflammatory signaling.

We demonstrate that pericyte‐conditioned media is sufficient to increase vessel formation in stromal‐endothelial cell co‐cultures, with results comparable to direct pericyte co‐cultures. Extracellular vesicles were isolated from this conditioned media, showing that pericytes release less but more homogenously sized vesicles with higher contents of pro‐angiogenic cytokines. In both the pericyte's conditioned media and EVs, IL‐6 was the cytokine with the highest fold change. Because of that, we decided to test the role of IL‐6 in the increased vasculogenesis by blocking it with the antibody siltuximab. The inhibition of IL‐6 dropped the vessel formation promoted by the pericytes to the same levels as stromal cell co‐cultures, proving that the pericyte's vasculogenic stimulation is dependent on this cytokine.

Overall, these results are relevant to understand the function of pericytes and stromal cells in vitro, and to improve current strategies for tissue‐engineered vascularization.

## 4 Experimental Section

### Cell Isolation and Culture

All human tissues were obtained following the Declaration of Helsinki after informed consent at RWTH Aachen University Hospital, Aachen, Germany. Stromal cells and pericytes were isolated from adipose tissue kindly provided by the Clinic for Plastic, Hand and Burns Surgery (RWTH Aachen University Hospital, ethics approval reference number EK197‐19). First, the adipose layer was separated from the dermis and manually minced until achieving a homogenous slurry. The minced fat was added to gentleMACS C‐Tubes (Miltenyi), mixed with the same volume of collagenase I (1 mg mL^−1^; Sigma–Aldrich), and placed in a gentleMACS Octo Dissociator to achieve complete homogenization and digestion at 37 °C with consecutive stirring steps for 15 min. The mixture was left for another 30 min at 37 °C in soft agitation on a roller mixer. The digested SVF was then filtered using a 100 µm cell strainer (Falcon), mixed with sterile PBS, and centrifuged at 300 g. The supernatant was removed and the pellet was resuspended in PBS for two more washing steps. To expand the stromal cells, one‐quarter of the SVF suspension was seeded in a cell culture‐plastic flask and cultured in Mesenpan media (PAN‐Biotech) supplemented with 2% (v/v) fetal bovine serum (FBS; Gibco) and 1% (v/v) antibiotic/antimycotic solution (ABM; Gibco).

The other part of the SVF is diluted 1:10 in Erythrocyte Lysis Buffer (PAN‐Biotech), briefly vortexed, and incubated at room temperature (RT) for 10 min. The mixture was washed another 3 times as previously described. The resulting pellet was resuspended in 10 µL anti‐CD146 magnetic microbeads (Miltenyi) and 90 µL blocking buffer (Miltenyi), and incubated for 15 min at 4 °C. The mixture was washed again three times to remove unattached magnetic microbeads. A MACS LS column (Miltenyi) was attached to a magnetic separator (Miltenyi) and prepared by rinsing it with PBS. The labeled cell suspension was added to the column and rinsed three times with Mesenpan to remove the CD146¯ cells. The column was then separated from the magnetic holder, filled with media, and pressed with a sterile plunger. The resulting CD146^+^ cell suspension was considered as pericytes and cultured as previously described in the same conditions as the stromal cells.

Umbilical cords were kindly provided by the Centralized Biomaterial Bank of the RWTH Aachen University (cBMB) and the Department of Gynecology and Perinatal Medicine according to RWTH Aachen University, Medical Faculty Ethics Committee (cBMB project number 323). Human umbilical vein endothelial cells (HUVECs) were isolated from umbilical cords using collagenase type I (Sigma–Aldrich, 400 U mL^−1^) to separate the cell layer surrounding the vein lumen. The detached cells were subsequently expanded in endothelial cell growth medium 2 (EGM2; Promocell) supplemented with 1% (v/v) ABM. The human umbilical cords were generously provided by the Clinic for Gynecology and Obstetrics (RWTH Aachen University Hospital) and cells were isolated within 24 h after delivery.

HUVECs, stromal cells, and pericytes were cryopreserved in liquid nitrogen using a freezing solution consisting of 80% (v/v) DMEM, 10% (v/v) FBS, and 10% (v/v) DMSO. Prior to the device experiment, HUVECs were thawed and expanded up to passage 3 in EGM2. Stromal cells and pericytes were cultured up to passage 4 in Mesenpan.

### Preparation of the Vascularization Assays in Microfluidic Devices

HUVECs, stromal cells and pericytes were detached by trypsin/EDTA (Pan‐Biotech) digestion. Cells were then suspended in tris‐buffered saline (TBS) solution containing thrombin (Sigma–Aldrich) and calcium chloride (CaCl_2_; Sigma–Aldrich). This mixture was combined with a fibrinogen solution (VWR) pre‐cooled on ice and either 10 µL were injected into the central channel of an idenTx 3 microfluidic chip (AIM Biotech) for the chip experiments or 350 µL were directly deposited into 24‐well plate wells for the bulk gels. The resulting gel contained 5 mg mL^−1^ fibrinogen, 3 IU mL^−1^ thrombin and 3.75 mM CaCl_2_.

For imaging after the preparation, cells were pre‐stained with Vybrant dyes (Invitrogen) by adding 5 µL of cell‐labeling solution to a 1 × 10^6^ cell mL^−1^ cell suspension in serum‐free cell media. The mixture was vortexed and incubated for 20 min at 37 °C. Then, cells were centrifuged at 300 g and washed with PBS three times to remove residual dye.

For the vasculogenesis chip set‐up, 10 × 10^6^ HUVECs mL^−1^ and 2 × 10^6^ supporting cells mL^−1^ were pre‐mixed in the injected solution. For the bulk vasculogenesis gel, 10 × 10^6^ HUVECs mL^−1^ and 5 × 10^6^ supporting cells mL^−1^ were added. For the angiogenesis chip set‐up, only 2 × 10^6^ supporting cells mL^−1^ were suspended in the polymerizing solution. In all cases, the fibrin gels were incubated for 20 min at room temperature and another 20 min at 37 °C and 5% CO_2_ in a humidified atmosphere to achieve complete polymerization of the fibrin. For the angiogenesis assay, the bottom channel was filled with a solution of 50 µg mL^−1^ fibronectin in EGM microvascular vessel 2 (EGM‐MV 2, PromoCell) immediately after the polymerization and incubated for 40 min at 37 °C. The channel was washed with EGM‐MV2 and a solution of 2 × 10^5^ HUVECs mL^−1^ was injected into the coated channel.

The reservoirs of all vasculogenesis chips were filled with EGM‐MV 2 supplemented with 1% (v/v) ABM, adding 100 µL to the bottom left well, 80 µL to the bottom right well, 60 µL to the top left well and 40 µL to the top right well, to achieve gravity‐driven flow both inside each of the channels and between them through the hydrogel. For angiogenesis, the same procedure was followed except for adding an additional 50 ng mL^−1^ VEGF (Peprotech) to the media in the bottom channels (opposite to the endothelialized channel and without cells) to attract endothelial cell migration. The chips were placed in humidified chambers with the bottom exposed to air, in a 5% CO_2_ and 37 °C atmosphere. The media was changed every 24 h for 10 days. For the bulk gels in 24‐well plates, 500 µL of EGM‐MV 2 was added and changed every 48–72 h. Three biological replicates (independent donors) were used in all cases.

For fixation, the medium was removed from the channels and substituted with ice‐cold methanol at ‐20 °C. All fibrin gels were incubated at 4 °C for 30 min and then washed three times with PBS.

### Live/Dead Staining

For live/dead staining, Calcein AM (Biozol) was added to EGM‐MV2 media to reach a final concentration of 2 µM. Media was fully removed from the chip channels and 100 µL of the Calcein‐media was added to each of the bottom wells, and 50 µL to each of the top wells. After 45 min of incubation, 0.2 and 0.1 µL of 2 mg mL^−1^ propidium iodide solution were added to the bottom and top channels respectively. The chips were imaged with an LSM 980 with an Airyscan 2 (Zeiss) confocal laser scanning microscope, creating Z‐stacks with a thickness of 150 µm with a step size of 3 µm.

### Immunofluorescence Imaging

The fixed chips were first stained with mouse anti‐CD31 (PECAM‐1, 1:100; Sigma–Aldrich) solved in antibody diluent made of 3% Bovine Serum Albumin (BSA) and 1% ABM for 48 h at 37 °C. Then, the samples were washed three times. Afterward, a mixture of Alexa Fluor® 594 goat anti‐mouse IgG (1:400, Invitrogen) and Phalloidin‐iFluor™ 488 Conjugate (Cayman Chemical) was added to the samples and incubated for 48 h at 37 °C. The chips were washed, stained with 0.4 µg mL^−1^ of a DAPI solution for 2 h at 37 °C, washed again, and stored at 4 °C until imaging. Microscopy was done with an LSM 980 with Airyscan 2 (Zeiss) confocal laser scanning microscope, creating stacks of the whole gel thickness, 150 µm, with a step size of 3 µm.

### Image Analysis and Quantification

The resulting composite was processed with Imaris 10 (Oxford Instruments) as described in Figure S2 (Supporting Information) to calculate vessel volume, distance to vessel, proliferation, and supporting cell sphericity. For the angiogenesis assay, in addition, the measure function of ImageJ was used to calculate the angiogenic sprouting length. The sproutings were considered tubular and homogenous enough to assess them as cylinders, approximating their diameter based on the data for volume and length. The number of branching points was manually counted.

### Cytokine Quantification

The media of the chip co‐cultures was saved and frozen on the last day of culture before fixation. Cytokine concentrations were measured using the angiogenesis panel I LEGENDPlex bead‐based immunoassay (BioLegend) for angiopoietin 1 (detection range: 3.4–120 000 pg mL^−1^), angiopoietin 2 (detection range: 18.8–100 000 pg mL^−1^), VEGF (detection range: 2.493–5000 pg mL^−1^), EGF (detection range: 0.51–10 000 pg mL^−1^), FGF‐2 (detection range: 0.1–440 000 pg mL^−1^), IL‐6 (detection range: 0.35–11 000 pg mL^−1^), IL‐8 (detection range: 1.52–10 000 pg mL^−1^), TNF (detection range: 1.921–9000 pg mL^−1^), PECAM‐1 (detection range: 13.2–100 000 pg mL^−1^) and PlGF (detection range: 0.2–110 000 pg mL^−1^). The assay was carried out following the product guidelines and measured by flow cytometry using the CytoFLEX LX (Beckman Coulter). The BioLegend LEGENDPlex software was used for analyses.

### TEM Imaging

The bulk fibrin gels were fixed in a 3% glutaraldehyde solution for at least 24 h. They were then rinsed in 0.1 M Soerensen's phosphate buffer (Merck) and fixed with 1% osmium tetraoxide (Carol Roth) in a 25 mM sucrose buffer (Merck). After a dehydration process with a series of increasing ethanol concentrations, the samples were immersed in propylene oxide, then in a 1:1 mixture of Epon resin (Serva) and propylene oxide for one hour, followed by a final one‐hour incubation in pure Epon resin. The samples were then embedded in pure Epon at 90 °C for two hours.

Ultrathin sections between 90 to 100 nm were obtained using an ultramicrotome (Reichert Ultracut S, Leica) equipped with a Diatome diamond knife. These sections were placed onto Cu/Rh grids (HR23 Maxtaform, Plano). To enhance contrast, the sections were stained with a 0.5% uranyl acetate and 1% lead citrate solution (both from EMS). The final observation was performed using a Zeiss Leo 906 (Carl Zeiss) transmission electron microscope, operated at an acceleration voltage of 60 kV.

### RNA Isolation, Sequencing, and Analysis

RNA isolation was performed using TRIzol (Invitrogen) and following the manufacturer's user guide. Briefly, cold TRIzol was added to the fibrin gel and incubated for 5 min. The mixture was then pipetted up and down until the gel was disagreggated, and stored at −20 °C until isolation. After thawing, chloroform was added to the sample, incubated for 3 min, and centrifuged for 15 min at 12 000 g at 4 °C to separate the phases. The top aqueous phase containing the RNA was carefully extracted and placed in a different tube. Then, isopropanol was added and the tubes were centrifuged again to precipitate the RNA. The supernatant was discarded, RNA pellet was resuspended in 75% ethanol and centrifuged again. This process was repeated three times to remove isopropanol leftovers from the mixture. Finally, the RNA pellet was air‐dried until no ethanol was left and resuspended in RNase‐free water.

3′mRNA‐Seq libraries were prepared using Lexogen QuantSeq 3′mRNA‐Seq v2 Library Prep Kit FWD with UDIs following the manufacturer's protocol. Prior to library preparation, the concentration of RNA was measured using the Promega Quantus Fluorometer. Additionally, the size distribution of the RNA was assessed using the Agilent TapeStation with an RNA ScreenTape. Quantification and quality assessment were repeated after library preparation, again using the Quantus fluorometer and the Agilent TapeStation with a High Sensitivity D1000 ScreenTape. Libraries were denatured, diluted, and loaded onto a NextSeq High Output v2.5 (75 cycles) flow cell. The 1% PhiX control library was spiked in to improve base calling accuracy. Single‐end sequencing was performed with 75 cycles on the Illumina NextSeq platform according to the manufacturer's instructions.

FASTQ files were generated using bcl2fastq (Illumina). To facilitate reproducible analysis, samples were processed using the publicly available nf‐core/RNA‐seq pipeline version 3.12^[^ ^53^ ^]^ implemented in Nextflow 23.10.0^[^ ^54^ ^]^ with minimal command. In brief, lane‐level reads were trimmed using Trim Galore 0.6.7^[^ ^55^ ^]^ and aligned to the human genome (GRCh39) using STAR 2.7.9a.^[^ ^56^ ^]^ Gene‐level and transcript‐level quantification was done by Salmon v1.10.1.^[^ ^57^ ^]^ Heatmap/dendrogram and volcano plot visualizations were performed using custom scripts in R version 4.3.2 using the DESeq2 v.1.32.0 framework.^[^ ^58^ ^]^

For the hallmark gene set analysis, Gene Set Enrichment Analysis (GSEA) was performed using clusterProfiler version 4.6.2. Pre‐ranked gene lists, generated from differential gene expression analysis, were input into clusterProfiler to identify enriched biological pathways.^[^ ^59^ ^]^ Enriched hallmark gene sets with an FDR q‐value < 0.05 were considered significant.

Gene network visualization and functional enrichment of GO pathways were done using the stringApp v2.0.3^[^ ^60^ ^]^ of CytoScape 3.10.1.^[^ ^61^ ^]^ For the gene networks, the top 200 genes with higher adjusted p‐values of each co‐culture type were plotted using the STRING visualization. Singletons and pairs were removed, and the remaining network was organized with the Prefuse Force Directed Layout. Then, enrichment data for all the signature genes of GO Biological Processes and GO Cellular Components was exported with a redundancy cutoff of “0.7” to remove nearly identical terms. The ten pathways with the highest adjusted FDR of each condition were plotted using GraphPad Prism 9.5.1.

### Media Conditioning

Pericytes and stromal cells (three biological donors for each) were seeded in T175 cell culture flasks and grown for 5–7 days at 37 °C with 5% CO_2_ in a humidified incubator in Mesenpan media, which was exchanged every 2–3 days. Once the cells reached 70%–80% confluency, they were incubated for 24 h with fresh EGM MV2 media, which was then removed, centrifuged for 10 min at 300 g, and mixed 1:1 with fresh EGM MV2 media to be used as conditioned media. Media incubated in flasks for 24 h at 37 °C and 5% CO_2_ was used as control. Glucose content was measured using a portable blood analyzer (EPOC). Conditioned media was prepared using three biological replicates (independent donors) for each of the two supporting cell types studied. In the respective chips, stromal cells and HUVECs from the same donors (which are independent for each cell type) were used to avoid any donor‐related differences.

### Extracellular Vesicle Isolation and Characterization

EVs were isolated from the previously described conditioned media via differential centrifugation as per established protocols.^[^ ^62^ ^]^ Briefly, conditioned media was initially centrifuged at 300 g for 10 min, followed by 2000 g for 20 min. The supernatant was then processed at 10 000 g for 40 min, filtered through a 0.2‐µm filter, centrifuged at 110 000 g for 70 min, washed with PBS, and centrifuged again at 110 000 g.

The size distribution and concentration of EVs were determined by NTA, using NanoSight NS300 (Malvern Instruments) equipped with a blue laser (488 nm, 70 mW). Parameters were adjusted to 12 in camera level, 5 in detection threshold, and 25 °C in temperature control to optimize performance. EV samples were diluted 1:100 in filtered PBS prior to NTA. The readings were acquired using the auto‐detection setting in 5 recording series of 60 s each. The data was analyzed with NTA 3.0 software (Malvern Instruments) and plotted using Excel 2016. TEM was performed as previously described.

For culturing the chips, the EVs were suspended in the same volume of fresh media as they were originally isolated from. The EV‐supplemented media was added and exchanged daily in stromal‐endothelial cell co‐cultures, following the vasculogenesis setup as previously described. EVs were isolated from the conditioned media of three biological replicates (independent donors) of each of the two supporting cell types studied. In the respective chips, stromal cells and HUVECs from the same donors (which are independent from each other) were used to avoid any donor‐related differences.

### Siltuximab Preparation

A glass vial containing 100 mg siltuximab (Sylvant, Recordati), was reconstituted in sterile cell culture‐grade water up to a concentration of 20 mg mL^−1^. Then, it was added to EGM MV2 media to achieve a final concentration of 100 ng mL^−1^. The siltuximab‐supplemented media was used to culture stromal‐endothelial cell co‐cultures in microfluidic chips with daily media changes for ten days, following the vasculogenesis setup as previously described.

### Statistical Analysis

All statistical analyses were performed and plotted using GraphPad Prism 9.5.1, except for the live/dead and the EV quantification which was plotted using Excel.

Normal distribution of the data was checked using the Shapiro‐Wilk test. For the comparison of variances, F‐test was performed. For two‐sample comparisons with normally distributed data, we used unpaired t‐tests. For a two‐sample comparison without normal distribution, the non‐parametric Mann‐Whitney test was used. For three‐sample comparisons with normally distributed data, one‐way ANOVA was performed with Tukey's correction to account for multiple comparisons. The Kruskal‐Wallis test was performed for non‐normally distributed data.

## Conflict of Interest

The authors declare no conflict of interest.

## Supporting information



Supporting Information

## Data Availability

The data that support the findings of this study are available from the corresponding author upon reasonable request.

## References

[1] G. Yang , B. Mahadik , J. Y. Choi , J. P. Fisher , Prog. Biomed. Eng. 2020, 2, 012002. 10.1088/2516-1091/ab5637 PMC8302186 34308105

[2] H. Zhao , J. C. Chappell , J. Biol. Eng. 2019, 13, 26. 30984287 10.1186/s13036-019-0158-3 PMC6444752

[3] P. Chandra , A. Atala , Clin. Sci. 2019, 133, 1115. 10.1042/CS20180155 31088895

[4] a) B. Zhang , M. Radisic , J. Thorac. Cardiovasc. Surg. 2020, 159, 2003. 31668537 10.1016/j.jtcvs.2019.08.128

[5] Y. Sun , S. Chen , X. Zhang , M. Pei , Arterioscler., Thromb., Vasc. Biol. 2019, 39, 1034. 31018663 10.1161/ATVBAHA.119.312425 PMC6531320

[6] P. Bourin , B. A. Bunnell , L. Casteilla , M. Dominici , A. J. Katz , K. L. March , H. Redl , J. P. Rubin , K. Yoshimura , J. M. Gimble , Cytotherapy 2013, 15, 641. 23570660 10.1016/j.jcyt.2013.02.006 PMC3979435

[7] S. M. Melief , J. J. Zwaginga , W. E. Fibbe , H. Roelofs , Stem Cells Transl. Med. 2013, 2, 455. 23694810 10.5966/sctm.2012-0184 PMC3673757

[8] A. E. S. Brooks , M. Iminitoff , E. Williams , T. Damani , V. Jackson‐Patel , V. Fan , J. James , P. R. Dunbar , V. Feisst , H. M. Sheppard , Front. Pharmacol. 2019, 10, 1695. 32153389 10.3389/fphar.2019.01695 PMC7044177

[9] A. Blocki , Y. Wang , M. Koch , P. Peh , S. Beyer , P. Law , J. Hui , M. Raghunath , Stem Cells Dev. 2013, 22, 2347. 23600480 10.1089/scd.2012.0415 PMC3749721

[10] Y. Wang , J. Xu , L. Chang , C. A. Meyers , L. Zhang , K. Broderick , M. Lee , B. Peault , A. W. James , npj Regen. Med. 2019, 4, 1. 30622740 10.1038/s41536-018-0063-2 PMC6323123

[11] Z. Wang , Q. Xu , N. Zhang , X. Du , G. Xu , X. Yan , Sig. Transduct. Target Ther. 2020, 5, 148. 10.1038/s41392-020-00259-8 PMC7421905 32782280

[12] B. Maniyadath , Q. Zhang , R. K. Gupta , S. Mandrup , Cell Metab. 2023, 35, 386. 36889280 10.1016/j.cmet.2023.02.002 PMC10027403

[13] A. I. Caplan , Stem Cells Transl. Med. 2017, 6, 1445. 28452204 10.1002/sctm.17-0051 PMC5689741

[14] D. G. Phinney , R. Hwa Lee , S. V. Boregowda , Stem Cells 2023, 41, 444. 36891977 10.1093/stmcls/sxad019 PMC10183967

[15] U. Galderisi , G. Peluso , G. Di Bernardo , Stem Cell Rev. Rep. 2022, 18, 23. 34398443 10.1007/s12015-021-10231-w PMC8365566

[16] S. Viswanathan , Y. Shi , J. Galipeau , M. Krampera , K. Leblanc , I. Martin , J. Nolta , D. G. Phinney , L. Sensebe , Cytotherapy 2019, 21, 1019. 31526643 10.1016/j.jcyt.2019.08.002

[17] a) C. M. Leung , P. de Haan , K. Ronaldson‐Bouchard , G.‐A. Kim , J. Ko , H. S. Rho , Z. Chen , P. Habibovic , N. L. Jeon , S. Takayama , M. L. Shuler , G. Vunjak‐Novakovic , O. Frey , E. Verpoorte , Y.‐C. Toh , Nat. Rev. Methods Primers 2022, 2, 33;

[18] M. Vila Cuenca , A. Cochrane , F. E. van den Hil , A. A. F. de Vries , S. A. J. Lesnik Oberstein , C. L. Mummery , V. V. Orlova , Stem Cell Rep. 2021, 16, 2159. 10.1016/j.stemcr.2021.08.003 PMC8452600 34478648

[19] S. Alimperti , T. Mirabella , V. Bajaj , W. Polacheck , D. M. Pirone , J. Duffield , J. Eyckmans , R. K. Assoian , C. S. Chen , Proc. Natl. Acad. Sci. USA 2017, 114, 8758. 28765370 10.1073/pnas.1618333114 PMC5565405

[20] N. Kosyakova , D. D. Kao , M. Figetakis , F. López‐Giráldez , S. Spindler , M. Graham , K. J. James , J. W Shin , X. Liu , G. T. Tietjen , J. S. Pober , W. G. Chang , npj Regen. Med. 2020, 5, 1. 31934351 10.1038/s41536-019-0086-3 PMC6944695

[21] A. Geevarghese , I. M. Herman , Transl. Res. 2014, 163, 296. 24530608 10.1016/j.trsl.2014.01.011 PMC3976718

[22] a) Y. Liu , J. Li , J. Zhou , X. Liu , H. Li , Y. Lu , B. Lin , X. Li , T. Liu , Micromachines. 2022, 13, 225;35208349

[23] R. Jayadev , D. R. Sherwood , Current Biology: CB 2017, 27, R207. 28324731 10.1016/j.cub.2017.02.006

[24] C. d. C. Picoli , A. Birbrair , Z. Li , Genes. 2024, 15, 126. 38275607 10.3390/genes15010126 PMC10815550

[25] R. G. Akwii , M. S. Sajib , F. T. Zahra , C. M. Mikelis , Cells 2019, 8, 471. 31108880 10.3390/cells8050471 PMC6562915

[26] P. Saharinen , L. Eklund , K. Alitalo , Nat. Rev. Drug Discov. 2017, 16, 635. 28529319 10.1038/nrd.2016.278

[27] P. Villar‐Fincheira , F. Sanhueza‐Olivares , I. Norambuena‐Soto , N. Cancino‐Arenas , F. Hernandez‐Vargas , R. Troncoso , L. Gabrielli , M. Chiong , Front. Mol. Biosci. 2021, 8, 641734. 33786327 10.3389/fmolb.2021.641734 PMC8004548

[28] E. Dejana , F. Orsenigo , J. Cell Sci. 2013, 126, 2545. 23781019 10.1242/jcs.124529

[29] S. Patel‐Hett , P. A. D'Amore , Int. J. Develop. Biol. 2011, 55, 353. 10.1387/ijdb.103213sp PMC4075038 21732275

[30] M. Fernández‐Chacón , I. García‐González , S. Mühleder , R. Benedito , Angiogenesis 2021, 24, 237. 34050878 10.1007/s10456-021-09793-7

[31] M. Hellström , L.‐K. Phng , J. J. Hofmann , E. Wallgard , L. Coultas , P. Lindblom , J. Alva , A.‐K. Nilsson , L. Karlsson , N. Gaiano , K. Yoon , J. Rossant , M. L. Iruela‐Arispe , M. Kalén , H. Gerhardt , C. Betsholtz , Nature 2007, 445, 776. 17259973 10.1038/nature05571

[32] A. A. Salybekov , A. K. Salybekova , R. Pola , T. Asahara , Int. J. Mol. Sci. 2018, 19, 3040. 30301174 10.3390/ijms19103040 PMC6213474

[33] J. J. Olsen , S. Ö.‐G. Pohl , A. Deshmukh , M. Visweswaran , N. C. Ward , F. Arfuso , M. Agostino , A. Dharmarajan , Clin. Biochemist. Rev. 2017, 38, 131. PMC5759160 29332977

[34] X. Wu , J. Zhou , D. Li , Front. Cell Dev. Biol. 2020, 8, 583325. 33072763 10.3389/fcell.2020.583325 PMC7533553

[35] a) A. D. Rusu , M. Georgiou , Open Biol. 2020, 10, 190278; 32070233 10.1098/rsob.190278 PMC7058937

[36] P. A. Guerrero , J. H. McCarty , in Physiologic and Pathologic Angiogenesis – Signaling Mechanisms and Targeted Therapy, InTech, London, United Kingdom 2017.

[37] M. C. Mendoza , E. E. Er , J. Blenis , Trends Biochem. Sci. 2011, 36, 320. 21531565 10.1016/j.tibs.2011.03.006 PMC3112285

[38] S. P. Didion , Int. J. Mol. Sci. 2017, 18.

[39] M. Patrikoski , B. Mannerström , S. Miettinen , Stem Cells Int. 2019, 2019, 1. 10.1155/2019/5858247 PMC6525805 31191677

[40] M. Mongiat , E. Andreuzzi , G. Tarticchio , A. Paulitti , Int. J. Mol. Sci. 2016, 17, 1822. 27809279 10.3390/ijms17111822 PMC5133823

[41] a) G. Bretones , M. D. Delgado , J. León , Bioch. Biophys. Acta 2015, 1849, 506; 10.1016/j.bbagrm.2014.03.013 24704206

[42] J. G. Carlton , H. Jones , U. S. Eggert , Nat. Rev. Mol. Cell Biol. 2020, 21, 151. 32034394 10.1038/s41580-019-0208-1

[43] K. Engeland , Cell Death Differ. 2022, 29, 946. 35361964 10.1038/s41418-022-00988-z PMC9090780

[44] a) S. Saxena , L. Zou , Mol. Cell 2022, 82, 2298; 35714587 10.1016/j.molcel.2022.05.004 PMC9219557

[45] A. Miyawaki‐Kuwakado , Q. Wu , A. Harada , K. Tomimatsu , T. Fujii , K. Maehara , Y. Ohkawa , Genes Cells 2021, 26, 530. 33987903 10.1111/gtc.12870

[46] U. Fiedler , M. Scharpfenecker , S. Koidl , A. Hegen , V. Grunow , J. M. Schmidt , W. Kriz , G. Thurston , H. G. Augustin , Blood. 2004, 103, 4150. 14976056 10.1182/blood-2003-10-3685

[47] S. J. Baek , S. K. Kang , J. C. Ra , Exp. Mol. Med. 2011, 43, 596. 21847008 10.3858/emm.2011.43.10.069 PMC3222821

[48] M. L. Da Alves Silva , A. R. Costa‐Pinto , A. Martins , V. M. Correlo , P. Sol , M. Bhattacharya , S. Faria , R. L. Reis , N. M. Neves , J. Tissue Eng. Regen. Med. 2015, 9, 714. 24155167 10.1002/term.1812

[49] E. Willms , H. J. Johansson , I. Mäger , Y. Lee , K. E. M. Blomberg , M. Sadik , A. Alaarg , C. I. E. Smith , J. Lehtiö , S. EL Andaloussi , M. J. A. Wood , P. Vader , Sci. Rep. 2016, 6, 22519. 26931825 10.1038/srep22519 PMC4773763

[50] T. Puchalski , U. Prabhakar , Q. Jiao , B. Berns , H. M. Davis , Clin. Cancer Res. 2010, 16, 1652. 20179212 10.1158/1078-0432.CCR-09-2581

[51] K. L. Whytock , Y. Sun , A. Divoux , G. Yu , S. R. Smith , M. J. Walsh , L. M. Sparks , iScience 2022, 25, 104772. 35992069 10.1016/j.isci.2022.104772 PMC9385549

[52] a) L. E. B. de Souza , T. M. Malta , S. K Haddad , D. T. Covas , Stem Cells Dev. 2016, 25, 1843; 27702398 10.1089/scd.2016.0109

[53] P. A. Ewels , A. Peltzer , S. Fillinger , H. Patel , J. Alneberg , A. Wilm , M. U. Garcia , P. Di Tommaso , S. Nahnsen , Nat. Biotechnol. 2020, 38, 276. 32055031 10.1038/s41587-020-0439-x

[54] P. Di Tommaso , M. Chatzou , E. W. Floden , P. P. Barja , E. Palumbo , C. Notredame , Nat. Biotechnol. 2017, 35, 316. 28398311 10.1038/nbt.3820

[55] F. Krueger , F. James , P. Ewels , E. Afyounian , M. Weinstein , B. Schuster‐Boeckler , G. Hulselmans , sclamons, Zenodo 2023, 10.5281/zenodo.5127898.

[56] A. Dobin , C. A. Davis , F. Schlesinger , J. Drenkow , C. Zaleski , S. Jha , P. Batut , M. Chaisson , T. R. Gingeras , Bioinformatics 2013, 29, 15. 23104886 10.1093/bioinformatics/bts635 PMC3530905

[57] R. Patro , G. Duggal , M. I. Love , R. A. Irizarry , C. Kingsford , Nat. Methods 2017, 14, 417. 28263959 10.1038/nmeth.4197 PMC5600148

[58] M. I. Love , W. Huber , S. Anders , Genome Biol. 2014, 15, 550. 25516281 10.1186/s13059-014-0550-8 PMC4302049

[59] A. Liberzon , C. Birger , H. Thorvaldsdóttir , M. Ghandi , J. P. Mesirov , P. Tamayo , Cell Systems 2015, 1, 417. 26771021 10.1016/j.cels.2015.12.004 PMC4707969

[60] N. T. Doncheva , J. H. Morris , J. Gorodkin , L. J. Jensen , J. Proteome Res. 2019, 18, 623. 30450911 10.1021/acs.jproteome.8b00702 PMC6800166

[61] P. Shannon , A. Markiel , O. Ozier , N. S. Baliga , J. T. Wang , D. Ramage , N. Amin , B. Schwikowski , T. Ideker , Genome Res. 2003, 13, 2498. 14597658 10.1101/gr.1239303 PMC403769

[62] a) C. Théry , S. Amigorena , G. Raposo , A. Clayton , Current Protocols in Cell Biology, 2006, Hoboken, NJ, Chapter 3, Unit 3.22; 10.1002/0471143030.cb0322s30 18228490

