# Supplementary material for: Pericytes Promote More Vascularization than Stromal Cells via an Interleukin‐6‐Dependent Mechanism in Microfluidic Chips
Source: Adv Sci (Weinh). 2025 Jan 30;12(14):2408131. doi: 10.1002/advs.202408131 (PMC11984840; doi:10.1002/advs.202408131)
Supplement: Supplementary file 1 — Supporting Information [file ADVS-12-2408131-s001.docx]

**Supplementary material**

**
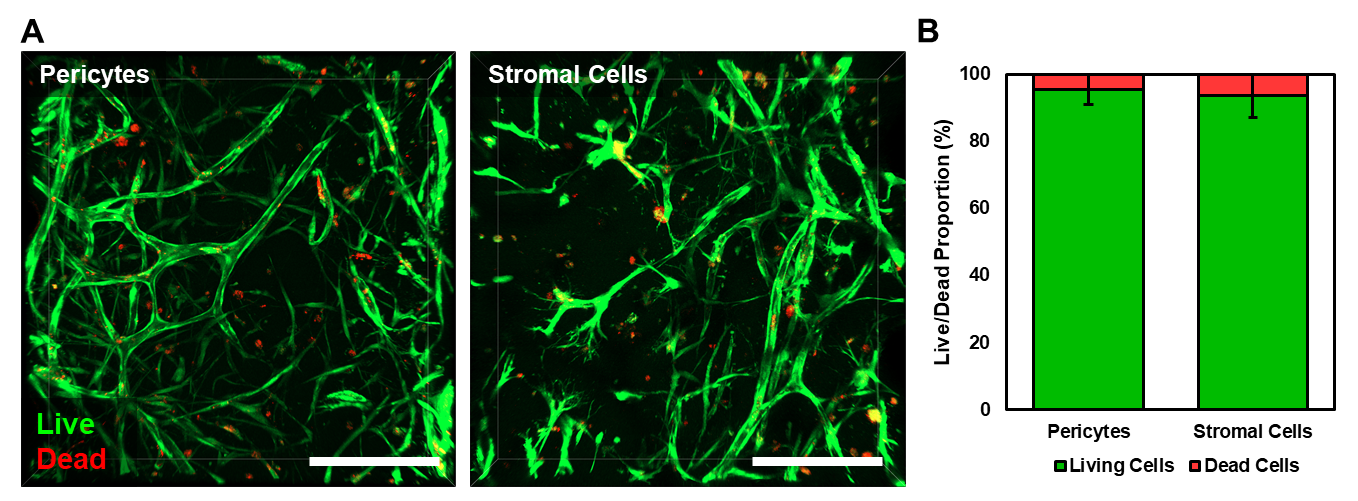
 Figure S1. Live/Dead staining of co-cultures in microfluidic chips. (A)** Confocal imaging of pericyte and stromal cell co-cultures with endothelial cells after ten days of incubation. The living cells were stained with calcein AM (green) and the dead cells with propidium iodide (red). Scale bar: 200 μm. **(B)** Quantification and comparison of the proportions of living and dead cells, showing no significant differences (n = 3, unpaired t-test, p < 0.05 is considered significant).


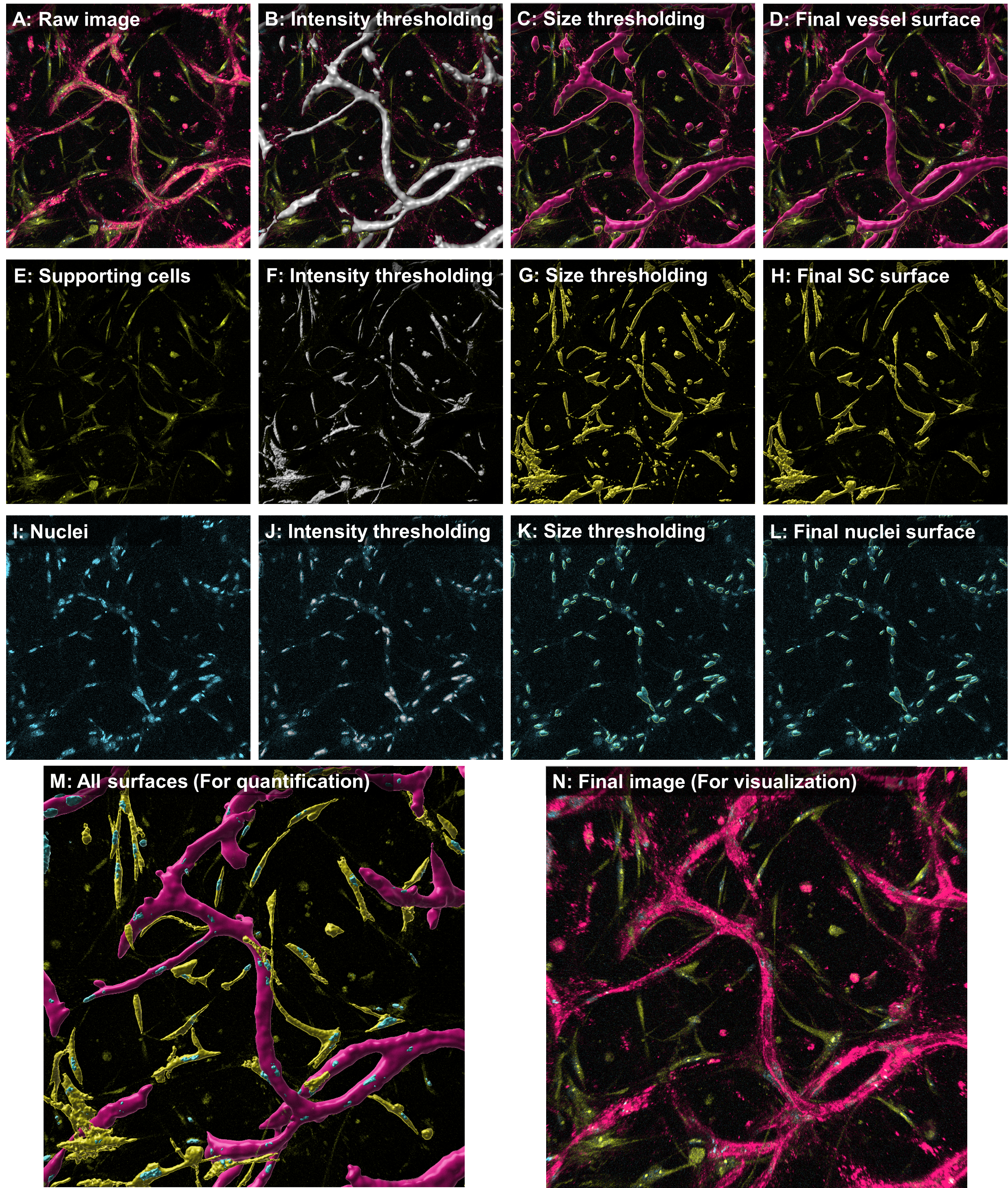


**Figure S2. Analysis pipeline of the confocal images. (A)** The raw image was loaded into Imaris 10. For visualization, the blue channel was changed to cyan, the green channel to yellow, and the red channel to magenta. **(B)** A new surface (3D object) was created based on the red channel (magenta), with a detail of 3 µm. The intensity threshold required for a voxel to be part of the vessel’s surface was defined in the range of 1,000 arbitrary units (AU). **(C)** A size threshold was defined at around 2,000 voxels to remove all the 3D surfaces generated that are smaller than a small aggregate of cells (1 voxel = 0.414 x 0.414 x 3). **(D)** Image showing the final result of the vessel’s surface creation. From this surface, the vessel volume is calculated. **(E)** The green channel, corresponding to the F-actin staining, marks both endothelial and supporting cells. This channel can be masked to remove all voxels within the vessel’s surface, leaving only the supporting cells. **(F)** A new 3D surface was created based on the green channel (yellow), with a detail of 1 µm (smaller detail because the supporting cells are smaller than the vessels). The intensity threshold required for a voxel to be part of the supporting cell’s surface was defined in the range of 1,000 AU. **(G)** A size threshold was defined to remove all the 3D surfaces generated that are smaller than one individual cell, around 2,500 voxels. **(H)** Image showing the final result of the supporting cell’s surface creation. From these 3D surfaces, each of the individual objects, Imaris can calculate the sphericity and the distance to the nearest vessel’s surface. **(I)** The blue channel (cyan) shows the nuclei of both endothelial and supporting cells. **(J)** A new 3D surface was created based on the blue channel (cyan), with a detail of 1 µm. The intensity threshold required for a voxel to be part of the nuclei’s surface was defined in the range of 1,000 AU. **(K)** A size threshold was manually defined to remove all the 3D surfaces generated that are smaller than one cell nucleus, around 300 voxels. **(L)** Image showing the final result of the nuclei’s surface creation. **(M)** Image showing all 3D surfaces created. The number of endothelial and supporting cells can be measured by calculating how many nuclei surfaces are within either the vessel’s or the supporting cells’ surfaces. **(N)** The final image that was used for visualization, was identical to the raw image except for the lack of green channel (yellow) signal in the area of the vessel.


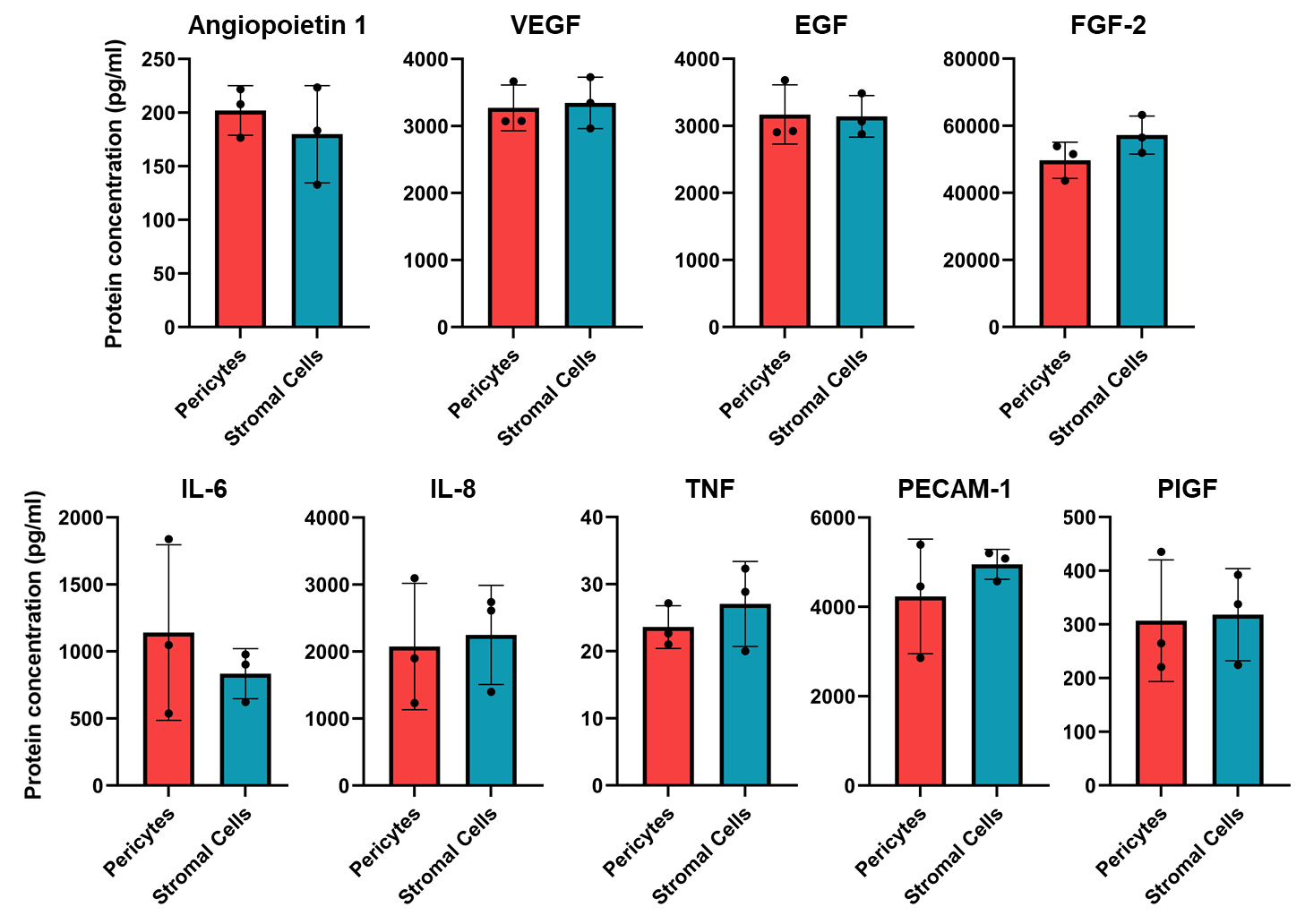


**Figure S3. Cytokine quantification in angiogenesis.** Angiopoietin 2 comparison was not included since its values were above the detection limit of the assay. Angiopoietin 1 (*p* = 0.4925), VEGF (*p* = 0.8096), EGF (*p* = 0.9332), FGF-2 (*p* = 0.1713), IL-6 (*p* = 0.4798), IL-8 (*p* = 0.8141), TNF (*p* = 0.4470), PECAM-1 (*p* = 0.4007) and PIGF (*p*= 0.8977).


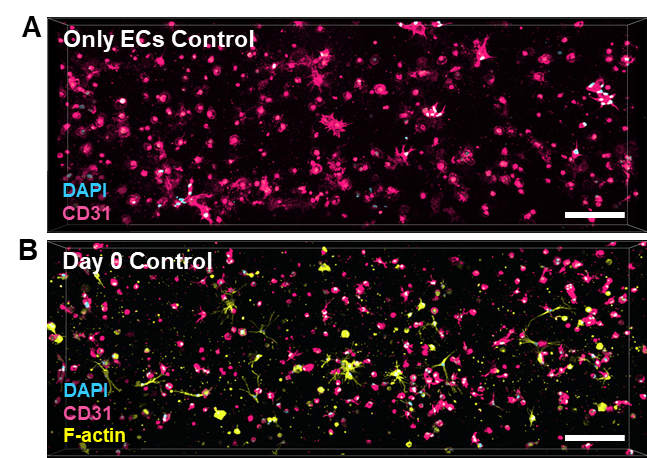


**Figure S4. Controls of the co-cultures. (A)** Mono-culture of HUVECs in fibrin gel after ten days, stained for the endothelial marker CD31 and nuclei (DAPI, blue). Most endothelial cells remain but do not form vessels. **(B)** Stromal-endothelial cell co-culture was fixed and stained on the day of preparation, 4 hours after the start of the polymerization. Scale bars: 200 μm.


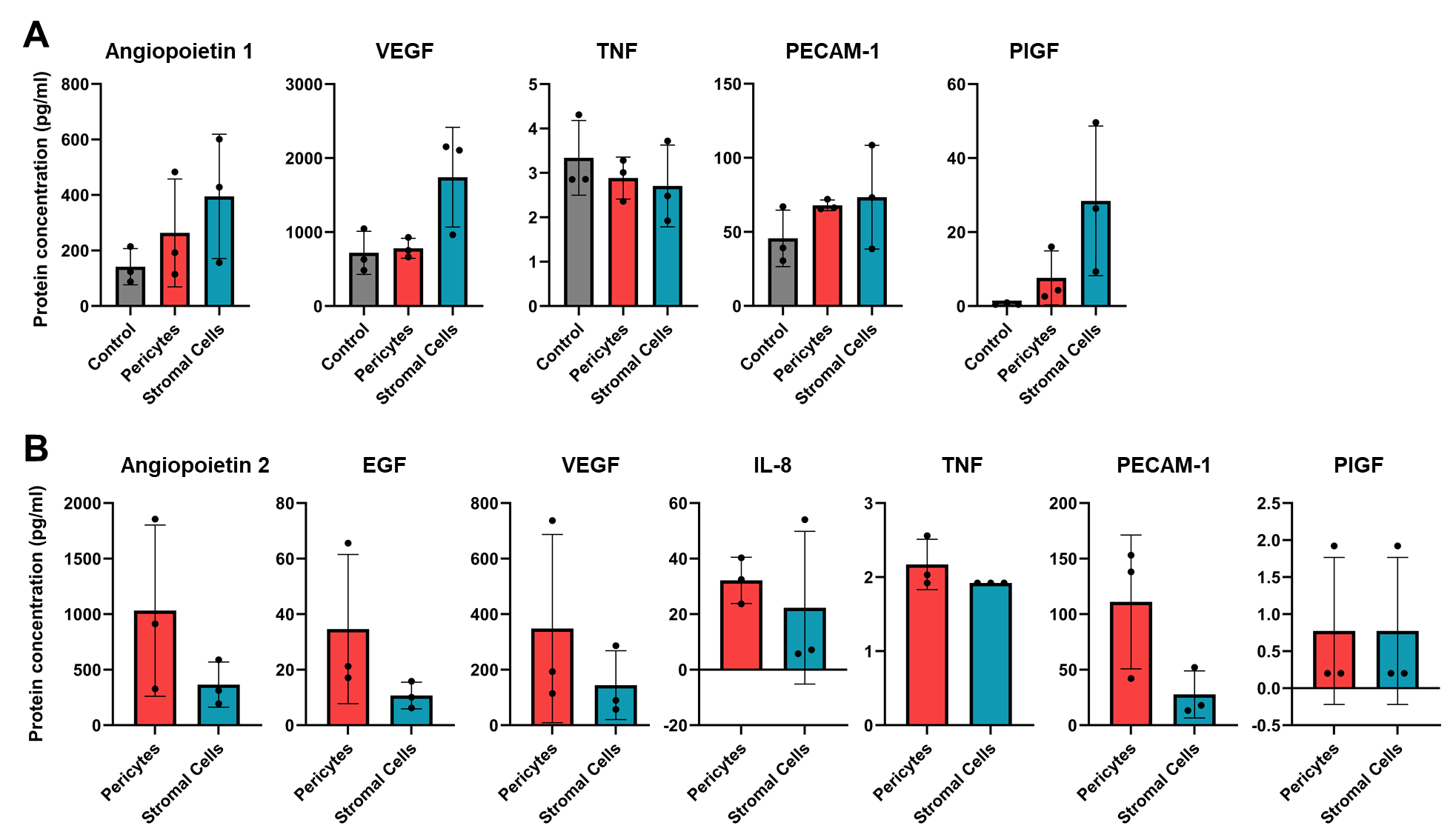


**Figure S5. Cytokines with no significant differences. (A)** Comparison of cytokines present in unconditioned media (Control) and pericyte/stromal cell conditioned media. Statistical values can be found in Table S2. **(B)** Comparison of EVs released by pericytes and stromal cells. Angiopoietin 2 (*p* = 0.2214), VEGF (*p* = 0.3830), EGF (*p* = 0.2039), IL-8 (*p* = 0.5849), TNF (*p* = 0.2732), PECAM-1 (*p* = 0.0867) and PlGF (*p* > 0.999) (n = 3, unpaired t-test, *p*< 0.05 is considered significant).

**Table S1. Comparison of the cytokine levels in the media of endothelial cell monocultures (OE) and co-cultures with pericytes (PC) or stromal cells (SC).** Statistical comparisons were performed using one-way ANOVA with Tukey’s post-hoc test for normally distributed data, or the Kruskal-Wallis test for non-normally distributed data (n = 3, p < 0.05 is considered significant).

|  | **Ang 1** | **Ang 2** | **VEGF** | **EGF** | **FGF-2** | **IL-6** | **IL-8** | **TNF** | **PECAM-1** | **PlGF** |
| --- | --- | --- | --- | --- | --- | --- | --- | --- | --- | --- |
| ***F*** | 0.4396 | 15.65 | 5.854 | 0.7824 | 26.16 | 7.385 | 3.678 | 3.769 |  | 7.449 |
| ***p* value** | 0.6635 | 0.0042 | 0.0389 | 0.499 | 0.0011 | 0.0241 | 0.0907 | 0.0871 |  | 0.0237 |
| ***H*** |  |  |  |  |  |  |  |  | 5.689 |  |
| ***p* value** |  |  |  |  |  |  |  |  | 0.0286 |  |
| **OE vs PC** | 0.6413 | 0.6495 | 0.1042 | 0.534 | 0.0018 | 0.0295 | 0.1469 | 0.1708 | 0.6991 | 0.0646 |
| **OE vs SC** | 0.8511 | 0.0047 | 0.0386 | 0.9953 | 0.002 | 0.9216 | 0.107 | 0.0918 | 0.0512 | 0.0242 |
| **PC vs SC** | 0.9227 | 0.0118 | 0.7191 | 0.5844 | 0.9877 | 0.0471 | 0.9667 | 0.8797 | 0.6991 | 0.7098 |

**Table S2. Comparison of cytokines levels in unconditioned media (Co) and media conditioned by pericytes (PC) or stromal cells (SC).** Statistical comparisons were performed using one-way ANOVA with Tukey’s post-hoc test for normally distributed data, or the Kruskal-Wallis test for non-normally distributed data (n = 3, p < 0.05 is considered significant).

|  | **Ang 1** | **Ang 2** | **VEGF** | **EGF** | **FGF-2** | **IL-6** | **IL-8** | **TNF** | **PECAM-1** | **PlGF** |
| --- | --- | --- | --- | --- | --- | --- | --- | --- | --- | --- |
| ***F*** | 1.571 | 19.55 | 5.292 |  | 46.95 | 20.59 | 6.077 |  | 1.216 | 4.084 |
| ***p* value** | 0.2828 | 0.0024 | 0.0474 |  | 0.0002 | 0.0021 | 0.0361 |  | 0.3602 | 0.0759 |
| ***H*** |  |  |  | 7.2 |  |  |  | 0.8 |  |  |
| ***p* value** |  |  |  | 0.0036 |  |  |  | 0.7214 |  |  |
| **Co vs PC** | 0.6877 | 0.003 | 0.9834 | 0.5391 | 0.0011 | 0.0017 | 0.3213 | 0.999 | 0.5039 | 0.7739 |
| **Co vs SC** | 0.2565 | 0.7708 | 0.0616 | 0.0219 | 0.0002 | 0.0731 | 0.0303 | 0.999 | 0.3673 | 0.0744 |
| **PC vs SC** | 0.6483 | 0.0057 | 0.0766 | 0.5391 | 0.1099 | 0.0254 | 0.2199 | 0.999 | 0.9557 | 0.1807 |
